# Characterisation of human pancreatic mesenchymal stromal cells in type 1 diabetes

**DOI:** 10.1007/s00125-025-06634-w

**Published:** 2025-12-21

**Authors:** Rebecca E. Dewhurst-Trigg, Jocelyn Atkins, Noel G. Morgan, Martin Eichmann, Sarah J. Richardson, Chloe L. Rackham

**Affiliations:** Exeter Centre for Excellence in Diabetes, Department of Clinical and Biomedical Sciences, University of Exeter, Exeter, UK

**Keywords:** Annexin A1, Beta cells, EADB, Inflammation, Islets of Langerhans, Mesenchymal stromal cells, nPOD, Pancreas, Type 1 diabetes

## Abstract

**Aims/hypothesis:**

Culture-expanded mesenchymal stromal cells (MSCs) reduce immune cell activation and improve islet functional survival. However, little is known about human pancreatic MSCs (pMSCs) in health or how they are altered in type 1 diabetes. Here, we determined the number, density and islet-protective phenotype of pMSCs in situ in individuals with and without type 1 diabetes.

**Methods:**

Multiplex immunohistochemistry was used to identify pMSCs (CD90^+^/CD105^+^/CD73^+^/CD31^−^/CD45^−^/CD34^−^) in human pancreas sections from 38 donors (Network for Pancreatic Organ Donors with Diabetes and Exeter Archival Diabetes Biobank). Donors were categorised as either <13 years at type 1 diabetes diagnosis (*n*=8) or ≥13 years at type 1 diabetes diagnosis (*n*=11) or were sex-matched individuals of similar age without diabetes. Consecutive sections were immunostained with antisera against insulin, glucagon and the established islet-protective and immunomodulatory factors annexin A1 (ANXA1) and indoleamine 2,3-dioxygenase 1. Whole-slide scans were acquired and pMSCs either inside or at the periphery (within 10 µm) of islets were quantified on an individual-islet basis. We identified 53,375 pMSCs and performed an analysis of 26,376 individual islets. Culture-expanded MSCs were exposed to cytokines and viability and proliferation were assessed by flow cytometry.

**Results:**

pMSCs were identified in situ in the human pancreas where they wrap around the islet periphery in an expected spindle-like morphology. ANXA1 was expressed by 33.2% of pMSCs and was expressed constitutively among individuals with or without diabetes. The density of both intraislet pMSCs and pMSCs within 10 µm of the islet periphery was increased for insulin-containing islets in individuals with type 1 diabetes compared with individuals without diabetes (*p*<0.001). pMSC density within 10 µm of the islet periphery was preferentially increased in individuals ≥13 years at type 1 diabetes diagnosis compared with individuals <13 years at type 1 diabetes diagnosis (*p*<0.001). pMSC density was reduced around insulin-deficient islets compared with insulin-containing islets in individuals with diabetes (*p*<0.001), consistent with an islet-protective role for pMSCs. Exposure of culture-expanded MSCs to an aggressive cytokine combination led to increased cell death and reduced proliferation.

**Conclusions/interpretation:**

pMSCs express ANXA1 constitutively, suggesting an islet-protective role in health. The density of pMSCs was increased around insulin-containing islets and lost around insulin-deficient islets in individuals with type 1 diabetes which aligns with this hypothesis. pMSC density at the periphery of insulin-containing islets was preferentially higher in individuals with later-onset type 1 diabetes, correlating with a less intense immune cell infiltration. The reduced ability of pMSCs to survive in the more intense proinflammatory environment around islets in younger-onset type 1 diabetes may contribute to the rapid rate of beta cell loss in these individuals.

**Graphical Abstract:**

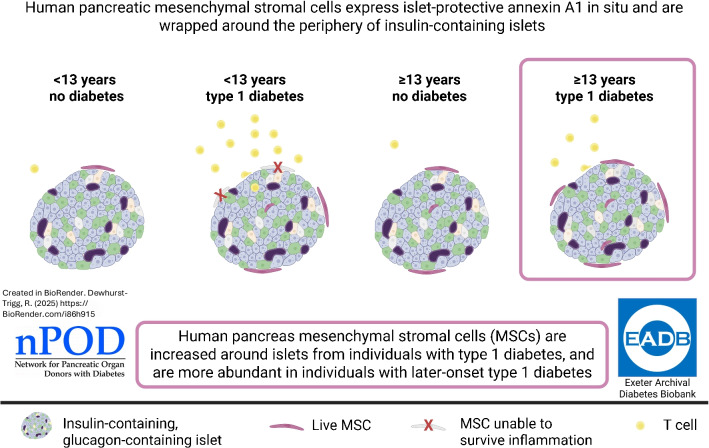

**Supplementary Information:**

The online version of this article (10.1007/s00125-025-06634-w) contains peer-reviewed but unedited supplementary material.


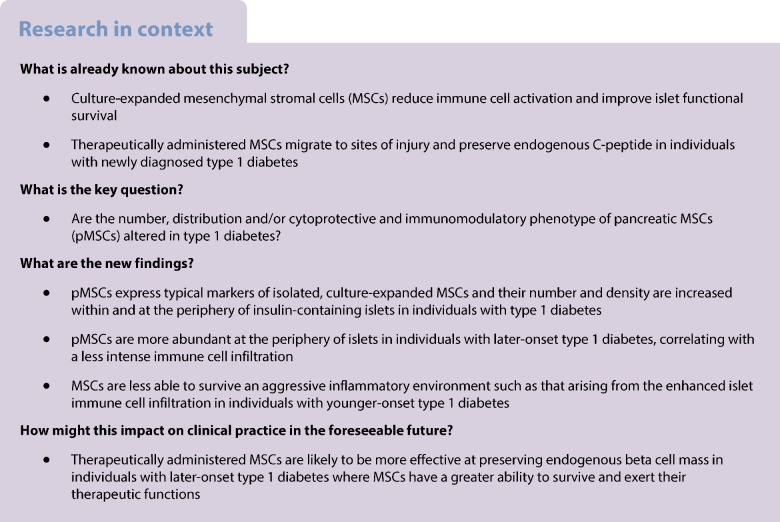


## Introduction

Mesenchymal stromal cells (MSCs) form the focus of attention in an increasing number of translational research fields, including diabetes [1–4], because of their strong immunomodulatory [5, 6] and regenerative [7–10] properties. We have begun to reveal the therapeutic mechanisms by which isolated and ‘culture-expanded’ MSCs (exogenous MSCs), derived from clinically relevant tissue sources (including bone marrow, adipose and pancreas), modulate islet function and survival [11–15], as well as immune cell activity [5, 16–18]. Intravenous infusion of culture-expanded MSCs preserves endogenous C-peptide in individuals with newly diagnosed type 1 diabetes [1, 2]. MSCs respond to specific cues within their microenvironment and migrate to sites of injury, including pancreatic islets [19], to exert their therapeutic actions.

Several studies have demonstrated that (culture-expanded) pancreas-derived MSCs have similar immunomodulatory and therapeutic potential to archetypal bone marrow MSCs [7, 8, 20–23]. These conclusions have been drawn based on studies using MSCs isolated initially from the pancreas, then expanded in culture and characterised according to their differentiation potential and surface expression of a panel of defined markers. Specifically, human culture-expanded MSCs, including those derived from pancreas [20, 24–26], are immunopositive for several surface antigens (CD73, CD90/Thy-1, CD105/endoglin), whilst they lack haematopoietic (CD34, CD45) and endothelial cell markers (CD31) [27, 28]. To our knowledge, a detailed immunohistological characterisation of human pancreatic MSCs (pMSCs) in situ has not been completed in individuals either with or without type 1 diabetes. Accordingly*,* little is known about the physiological roles of pMSCs or how these may be altered in type 1 diabetes.

No single phenotypic marker can distinguish MSCs from other stromal or perivascular cell types. Multiplex immunohistochemistry methods therefore offer a robust approach for high-resolution, simultaneous detection of multiple antigens within a single tissue section and provide a suitable methodology for the detection and characterisation of pMSCs in situ*.* We have used multiplex immunohistochemistry to identify pMSCs (CD73^+^, CD90^+^, CD105^+^, CD31^−^, CD34^−^, CD45^−^) in human pancreas sections from 38 donors (Network for Pancreatic Organ Donors with Diabetes [nPOD] and Exeter Archival Diabetes Biobank [EADB]) with short-duration type 1 diabetes (≤2 years) or similarly aged donors without type 1 diabetes. Multiplex immunohistochemistry is particularly advantageous in studies using rare and finite tissue resources, such as pancreas tissue available for research in young individuals with short-duration type 1 diabetes. To maximise insights into type 1 diabetes disease mechanisms and progression, the conservation of these valuable and irreplaceable tissue samples is essential across the research community. Accordingly, this study employed the Opal-tyramide signal amplification immunostaining method, whole-slide scanning (PhenoImager HT) and the quantitative digital pathology platform HALO to maximise data generation from each tissue section. This approach enabled the simultaneous detection of six distinct tissue antigens per section and data analysis at the individual-islet level. The defined 6-plex panel of MSC markers is comparable to that expressed by MSC populations from different tissue sources, including pancreas [20, 23], which have been shown to improve islet functional viability in preclinical models of type 1 diabetes [23], and to preserve C-peptide in individuals with newly diagnosed type 1 diabetes [1, 2].

Understanding how the number, distribution and islet-protective phenotype of pMSCs are altered in type 1 diabetes is of critical importance as these cells become a therapeutic option for intervention in type 1 diabetes pathogenesis and progression.

## Methods

### Human tissue

Formalin- or mercuric chloride-fixed, paraffin-embedded pancreas tissue sessions were obtained from within the EADB and nPOD collections. Ethical permission from the West of Scotland Research Ethics Committee (ref: 20/WS/007; Integrated Research Application System Project ID: 283620) and from the nPOD tissue prioritisation committee, University of Florida (Project ID: 24-013) was obtained for the use of EADB and nPOD samples, respectively.

Analyses were performed using donors with short-duration (≤2 years) type 1 diabetes who were <13 years at type 1 diabetes diagnosis or ≥13 years at type 1 diabetes diagnosis. Control individuals were sex-matched donors without type 1 diabetes of similar age and BMI. The cut-off for defining two groups according to age of diagnosis has been informed by previous studies in which individuals with type 1 diabetes were categorised as type 1 diabetes endotype 1 (T1DE1) or type 1 diabetes endotype 2 (T1DE2), according to distinct histological phenotypes (CD20Hi/Lo) and extent of proinsulin processing [29]. Individuals diagnosed at <13 years often present with a more aggressive disease associated with increased immune cell infiltration and greater loss of residual beta cells in the pancreas [29, 30]. We acknowledge that the current report does not directly categorise individuals with type 1 diabetes according to these distinct histological phenotypes to avoid unnecessary tissue use. Instead, it used an age at diagnosis cut-off of 13 years to align with the T1DE2 categorisation on the understanding that most younger donors will then be defined as T1DE1. Thus, for the current report, our type 1 diabetes subcategorisation is not absolutely endotype-specific but, as a surrogate, it stratifies specific groups of individuals by virtue of their age at diagnosis. Individuals were included in the current study from the EADB (*n*=3, <13 years at type 1 diabetes diagnosis; *n*=3, <13 years without diabetes; *n*=3, ≥13 years at type 1 diabetes diagnosis; *n*=3, ≥13 years without diabetes) and nPOD (*n*=5, <13 years at type 1 diabetes diagnosis; *n*=5, <13 years without diabetes; *n*=8, ≥13 years at type 1 diabetes diagnosis; *n*=8, ≥13 years without diabetes) collections. Individual donor characteristics are detailed in electronic supplementary material (ESM) Table 1. Age was similar between individuals <13 years with type 1 diabetes and without diabetes [mean ± SEM; 6.80±1.54 years vs 6.13±1.01 years, respectively; *t*(14)=0.3656, *p*=0.72] and between individuals ≥13 years with type 1 diabetes and without diabetes [19.35±1.44 years vs 17.75±1.34 years, respectively; *t*(20)=0.8085, *p*=0.43]. Donors of European descent (self- or family-reported ethnicity taken from medical records) were selected to ensure that any alterations in pMSCs were not due to the emerging evidence of heterogeneity of type 1 diabetes between ethnic groups (reviewed in [31]). For nPOD donors where information was available, pancreas sections were taken from the pancreas tail and block numbers were matched for individuals with and without diabetes.

### Multiplex immunohistochemistry

We used 4 μm human pancreas, tonsil, intestine and placenta sections from the EADB collection for initial antibody and subsequent Opal 6-plex panel optimisation. The Opal multiplex panel optimisation and protocol are summarised in ESM Fig. 1. The final 6-plex panels to identify pMSCs and islet hormone and MSC islet-protective factors are detailed in ESM Tables 2 and 3, respectively.

Following tissue rehydration, multiplex immunostaining was performed using the Opal 6-plex manual detection kit (Akoya Biosciences). Heat-induced epitope retrieval (HIER) was performed for 20 min using a pressure cooker. HIER buffers were made in-house containing either 0.01 mol/l citrate (Merck), pH 6, or 10 mmol/l TrisBase (Merck) and 1 mmol/l EDTA (Merck), pH 9, and were specific to each antigen (see ESM Tables 2 and 3 for details).

One section per donor was immunostained with antibodies against CD90, CD105, CD73, CD31, CD34 and CD45 to identify pMSCs. A serial section was then immunostained with antibodies against CD90, indoleamine 2,3-dioxygenase 1 (IDO1), annexin A1 (ANXA1), CD45, insulin and glucagon to identify islets and MSC-derived islet-protective factors. Antigens were visualised using the Opal fluorophores: 480, 520, 570, 620, 690 and 780, and sections were counterstained with DAPI (Invitrogen). Once the immunostaining was complete, sections were mounted using ProLong Diamond Antifade mounting medium (Invitrogen).

### Image acquisition and processing

Slides were scanned at ×40 magnification using the MOTiF workflow on a PhenoImager HT (v1.0.11, Akoya Biosciences) within 2 weeks of mounting the tissue. Exposure times were determined as the mean time following auto-exposure at several positions across the tissue. Exposure times were set separately for each tissue biobank (EADB and nPOD) and remained consistent across all sections within each biobank. Spectral unmixing was performed in inForm (v2.6, Akoya Biosciences) using the Akoya synthetic library and Phenochart (v1.1.0, Akoya Biosciences). Autofluorescence subtraction was adjusted individually for sections from the EADB due to variations in innate tissue autofluorescence across sections. Autofluorescence subtraction remained consistent for all nPOD sections where autofluorescence remained similar across sections.

### Image quantification

Image quantification was performed in HALO (v3.6, Indica Labs). Serial sections from each donor were registered and fused. Serial section registration was manually verified and adjusted where required to ensure optimal alignment. Then, a random forest classifier was used to identify pancreas tissue and islets (based on positive insulin and/or glucagon staining) that were larger than the estimated area of at least one cell (170 µm^2^) [32]. Islet annotations were generated from the classifier and exported from HALO as a .GeoJSON file. All islet annotations were simultaneously expanded outwards in QuPath (v0.5.1, [33]) by 10 µm or until they reached another expanding annotation to identify the islet periphery. This avoided any overlapping annotation areas, which ensured that cells were not counted twice during individual-islet analysis. Each islet annotation was assigned a unique identifier in Python (v3.12, https://www.python.org) and then re-imported into HALO for analysis. Quantitative analysis of cell populations was performed using HighPlex FL (v4.2.14, HALO, Indica Labs). First, cells were identified using DAPI and positive cytoplasmic and nuclear staining thresholds were set for all antigens. Staining thresholds were adjusted for each donor individually. Next, cell phenotypes were determined and pMSCs were identified as CD90^+^, CD73^+^, CD105^+^, CD31^−^, CD34^−^, CD45^−^ cells. HighPlex FL analysis was run in batches across all annotation layers to give individual-islet data, including intraislet data and islet periphery (10 µm outside of the islet) data (calculated by expanded islet minus islet annotation). Donor blinding was not possible for the quantitative analysis due to the inclusion of insulin immunostaining in the Opal panel.

Prior to analysis, individual islets were categorised into one of three categories based on their endocrine cell composition: insulin-positive glucagon-negative (INS^+^GLU^−^), insulin-positive glucagon-positive (INS^+^GLU^+^) or insulin-negative glucagon-positive (INS^−^GLU^+^). Islets were categorised as positive for insulin or glucagon if they contained one or more positive cells for the respective marker. Islets were further categorised by size by calculating the base-2 logarithm of the ratio between the measured islet area and the estimated individual cell area (170 µm^2^), then rounded down to the nearest integer [32]. This logarithmic scale provides fine detail for the high number of small islets and larger bins for the lower number of large islets. Representative images of islets from each size category are shown in ESM Fig. 2.

**Fig. 1:**
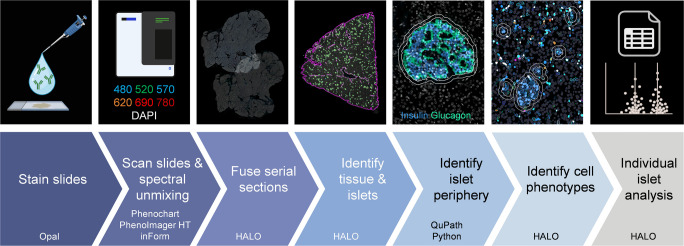
Optimised workflow for the generation of quantitative data, on an individual-islet basis, for the number and density of pMSCs associated with islets of different endocrine cell compositions and size. Serial sections were immunostained with our optimised 6-plex Opal panel to identify pMSCs or islet hormones and MSC-derived islet-protective factors. Slides were scanned at ×40 magnification using the MOTiF workflow on a PhenoImager HT and multiplex images were spectrally unmixed. Unmixed whole-slide scans were loaded into HALO and serial sections registered and fused. A random forest classifier was used to identify glass, tissue and islets that were more than 170 µm^2^ (magenta annotation shows pancreas outline, and green annotations show islets). Islet annotations were imported into QuPath and expanded by 10 µm or until they reached another expanding annotation (white annotations show the islet periphery, and the islet annotation expanded by 10 µm). Positive staining thresholds were set for all antigens and cell phenotypes identified. Data analysis was performed on each individual islet and expanded islet annotation (islet periphery data determined by expanded islet minus islet data). Donor IDs: nPOD 6271, EADB 12426, EADB E386, nPOD 6488. Figure created in BioRender

Our optimised workflow for the generation of quantitative data on an individual-islet basis for the number, density and islet-protective phenotype of pMSCs associated with islets of different endocrine cell compositions and size is shown in Fig. 1.

### MSC viability and proliferation following cytokine exposure in vitro

Human adipose tissue-derived MSCs (StemPro Human Adipose-derived Stem Cells, positive for human MSC markers: CD73^+^, CD90^+^, CD105^+^, and negative for CD31^−^ and CD45^−^, in accordance with our defined Opal 6-plex panel of MSC markers, female donor 51 years, Gibco; verified mycoplasma-free in-house, MycoAlert Plus, Lonza Biosciences) were labelled with 0.5 mmol/l CellTrace Far Red (Invitrogen) according to the manufacturer’s recommendations to enable quantification of proliferation and seeded into 35 mm wells (Sarstedt) at 75,000 cells/well. MSCs were cultured in DMEM supplemented with 10% (vol/vol) FBS, 100 U/ml penicillin, 100 µg/ml streptomycin and 2 mmol/l l-glutamine (all reagents from Merck). MSCs were cultured at 37°C in a humidified environment containing 5% CO_2_. MSCs were left to adhere overnight and then cultured for 3 or 7 days in either normal medium (no cytokine control) or normal medium containing: IFN-α alone; IFN-γ + IL-1β; or IFN-γ + IL-1β + TNF-α. The concentration of each cytokine was: IFN-α (1000 U/ml, PBL Assay Science), IFN-γ (1000 U/ml, PeproTech), IL-1β (50 U/ml, PeproTech) and TNF-α (1000 U/ml, PeproTech). Medium was refreshed on day 3 of cytokine exposure. To prepare samples for analysis, supernatant was collected, and cells were detached with Accutase (Merck). Cells were washed in cold PBS then 0.5% FBS in PBS and resuspended in 600 µl of 0.5% FBS in PBS. To quantify MSC viability, one drop of Sytox Green (Invitrogen) was added 5 min before acquisition by flow cytometry (Attune, ThermoFisher Scientific). Flow cytometry analysis of MSC viability and proliferation was performed in FlowJo (v10.8.1, BD Life Sciences) using the gating strategy shown in ESM Fig. 3. The percentage of divided cells was calculated using the FlowJo proliferation algorithm to determine proliferation of CellTrace-labelled cells.

### MSC gene expression of islet-protective factors

Human adipose tissue-derived MSCs were seeded into 35 mm wells at 100,000 cells/well and exposed to cytokines for 24 h, as described above. Total RNA was extracted from MSCs using QIAshredder, RNeasy and RNase-free DNase kits (Qiagen) and 500 ng of RNA (measured by nanodrop, ThermoFisher Scientific) was reverse transcribed into cDNAs using high-capacity reverse transcription kits (Applied Biosystems). Quantitative PCR (qPCR) was performed using QuantiTect primer assays (ESM Table 4) and QuantiNova SYBR Green (Qiagen). Data were acquired using a QuantStudio 12K Flex RT-qPCR system (ThermoFisher Scientific). Melt curve analysis was performed to confirm the specificity of each amplicon. Gene expression levels of target genes, *IDO1* and *ANXA1*, were normalised against the geometric mean of four internal reference genes: *ACTB*, *GAPDH*, *PPIA* and *HPRT1*. Relative changes in the expression of target genes between cytokine conditions were expressed relative to the no cytokine condition and calculated by the ${2}^{-\Delta \Delta {\mathrm{C}}_{t}}$ method.

### Statistical analysis

Quantitative data on an individual-islet basis were generated from whole-slide scans using HALO. Raw data were exported to Excel (v2508, Microsoft 365), and descriptive statistics (including mean, SD, SEM and *n*) generated with R (v4.4.0 https://www.R-project.org/ [34]. Preliminary code generation was facilitated using an artificial intelligence language model (ChatGPT, vGPT-4o). All generated code was reviewed, validated and refined manually to ensure accuracy and suitability for this study. Statistical analysis and graphical representation of these data were performed in GraphPad Prism (v10, GraphPad) and included unpaired *t* test, one-way ANOVA and two-way ANOVA, as indicated in each figure legend. Where appropriate, post hoc analyses for ANOVAs were performed, and were adjusted for multiple comparisons with the Bonferroni correction. We report *p* values as: ***p≤0.001; **p≤0.01; *p≤0.05. Where individual-islet data are shown, mean ± SEM data are shown for clarity due to the large number of data points and completion of statistical analysis using descriptive statistics in GraphPad. Where possible, individual data points are shown. Where data are separated by islet bin size, categories with <5 islets in at least one group were excluded from analysis. All data are reported as mean ± SEM.

## Results

### Islet endocrine cell composition among individuals with and without type 1 diabetes

A total of 26,376 individual islets were identified across the 38 individuals with or without type 1 diabetes included in this study. The number and proportion of islets comprising each endocrine cell composition (INS^+^GLU^−^, INS^+^GLU^+^ and INS^−^GLU^+^) are shown as a mean per group in Table 1 and for individual donors in ESM Table 5. The density and proportion of islets comprising each endocrine cell composition, and separated by islet bin size, are shown in ESM Fig. 4. These data highlight a marked reduction in both the density and proportion of small-to-medium insulin-containing (INS^+^GLU^−^ and INS^+^GLU^+^) islets for individuals with type 1 diabetes. This is consistent with recent findings that smaller insulin-containing islets are largely absent at onset of type 1 diabetes, and those islets that retain insulin are larger in size [35]. Our 2D data from individuals without diabetes align with recent observations made in adult pancreas using 3D imaging technologies, which highlight that a significant proportion (approximately 50%) of insulin-containing islets are devoid of glucagon [36].

**Table 1:** Mean number and percentage of islets comprising different endocrine cell compositions among individuals with and without type 1 diabetes from the EADB and nPOD collections

Group	Islets of each endocrine cell composition (*n*)			Total number of islets	Islets of each endocrine cell composition (%)		
	INS^+^GLU^−^	INS^+^GLU^+^	INS^−^GLU^+^		INS^+^GLU^−^	INS^+^GLU^+^	INS^−^GLU^+^
<13 y T1D	11±7**^a^	29±10***^a^	291±85	331±85	3.47±2.30***^a^	10.54±3.53***^a^	85.99±5.62***^a^
<13 y ND	359±96	407±60	40±11	807±138	42.70±5.02	51.76±4.03	5.54±1.88
≥13 y T1D	27±12***^b^	87±16***^b^	490±143*^b^	604±145	7.15±2.81***^b^	22.93±4.59**^b^	69.92±6.40***^b^
≥13 y ND	432±86	413±84	122±41	966±201	46.43±3.35	43.95±3.16	9.62±1.65

Data are mean ± SEM. Individual donor statistics are reported in the ESM

Individuals included in these data are: <13 years at type 1 diabetes diagnosis, *n*=8; <13 years without diabetes, *n*=8; ≥13 years at type 1 diabetes diagnosis, *n*=11; ≥13 years without diabetes, *n*=11

Ordinary one-way ANOVA for each column with post hoc tests adjusted for multiple comparisons with Bonferroni correction. Post hoc comparisons within column:

^a^Compared with <13 years without diabetes

^b^Compared with ≥13 years without diabetes

^***^p≤0.001; **p≤0.01; *p≤0.05

<13 y ND, <13 years without diabetes; <13 y T1D, <13 years at type 1 diabetes diagnosis; ≥13 y ND, ≥13 years without diabetes; ≥13 y T1D, ≥13 years at type 1 diabetes diagnosis

In line with the classification of individuals with type 1 diabetes into two groups based on age at diagnosis and therefore aggressiveness in beta cell destruction, the proportion of INS^+^GLU^+^ islets tended to be higher in individuals ≥13 years at diagnosis of type 1 diabetes compared with individuals <13 years at diagnosis of type 1 diabetes [mean ± SEM; 22.93±4.59% vs 10.54±3.53%, respectively; unpaired *t* test: *t*(17)=2.002, *p*=0.06]. This aligns with previous observations demonstrating a higher proportion of insulin-containing islets in individuals with T1DE2 compared with T1DE1 [29, 30]. The identification of islets according to the three defined endocrine cell compositions allowed us to investigate whether pMSCs were preferentially associated with islets of a particular endocrine cell composition.

### An extensive 6-plex panel is required to accurately identify pMSCs in situ

MSCs share phenotypic markers with endothelial cells, pericytes, fibroblasts and stellate cells. Thus, no single phenotypic marker can distinguish MSCs from other stromal or perivascular cell types. To ensure the most efficient use of rare and finite human pancreatic tissue, we initially evaluated whether a simplified 3-plex panel (CD90^+^, CD105^+^, CD31^−^) could accurately phenotype pMSCs in situ, or whether a more comprehensive 6-plex panel was required. CD90 and CD105 are widely used as positive human MSC markers; however, they are also expressed by endothelial cells. To address this, we included the endothelial cell marker CD31 as a negative MSC marker, in line with the International Society for Cell and Gene Therapy (ISCT) minimal criteria for MSC characterisation. CD90 and CD105 are two of the three (CD90, CD105 and CD73) positive human MSC markers outlined by the ISCT for defining human MSC identity [27, 28]. Accordingly, the 6-plex panel included CD73 as an additional positive MSC marker, as well as CD34 (endothelial cell and haematopoietic stem cell marker) and CD45 (pan-immune cell marker) as additional negative MSC markers (CD73^+^, CD90^+^, CD105^+^, CD31^−^, CD34^−^, CD45^−^).

Whole pancreas MSC density across EADB and nPOD donors was markedly overestimated when phenotyped using a core 3-plex (CD90^+^, CD105^+^, CD31^−^), compared with the comprehensive 6-plex panel (CD73^+^, CD90^+^, CD105^+^, CD31^−^, CD34^−^, CD45^−^) [mean ± SEM; 123±19 vs 14±3 MSCs/mm^2^, respectively; *t*(74)=5.647, *p*<0.001]. This validates the importance of characterising pMSCs using a 6-plex panel, to ensure cell types of distinct but similar phenotypes are excluded from the pMSC population. Using the comprehensive 6-plex panel, a total of 53,375 pMSCs (CD73^+^, CD90^+^, CD105^+^, CD31^−^, CD34^−^, CD45^−^) were identified across the 38 individuals included in this study. The pMSC population defined by the comprehensive 6-plex panel was further analysed to determine whether their number, density and/or islet-protective phenotype was altered in type 1 diabetes.

### Whole pancreas pMSC density is similar in individuals with and without type 1 diabetes

The density of pMSCs across the total pancreas area including acinar, islets, ducts and vessels was variable between individuals both with and without type 1 diabetes (range 0–63 and 1–94 pMSCs/mm^2^, respectively), as expected given the established migratory capacity of MSCs [19]. Together, the density of pMSCs across the whole pancreas area was similar for individuals with and without type 1 diabetes [14.70±3.98 vs 14.07±5.17 pMSCs/mm^2^, respectively; *t*(36)=0.0966, *p*=0.92]. When separated by age, total pancreas pMSC density was similar between individuals with or without type 1 diabetes [one-way ANOVA; *F*(3, 34)=0.9442, *p*=0.43]. Representative spatial plots showing the distribution of MSCs across the endocrine and exocrine pancreas are shown in ESM Fig. 5.

### Detection of pMSCs in proximity to pancreatic islets

**Fig. 2:**
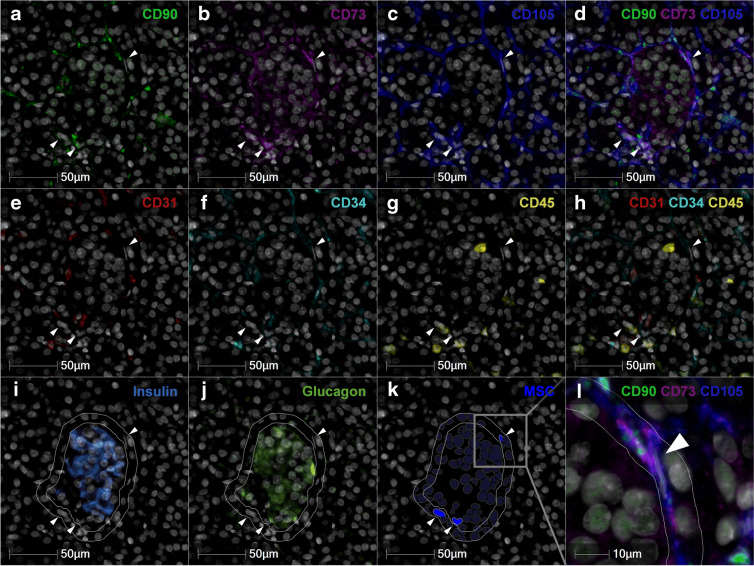
Phenotype and morphology of pMSCs at the islet periphery. pMSCs (shown by white arrows, **a**–**l**) at the islet periphery were identified in HALO as CD90^+^ (**a**), CD73^+^ (**b**), CD105^+^ (**c**); overlay of positive markers (**d**); CD31^−^ (**e**), CD34^−^ (**f**), CD45^−^ (**g**); overlay of negative markers (**h**). Islets were identified by insulin (**i**) and glucagon (**j**) immunostaining (inner white annotation) and the islet annotation expanded by 10 µm or until another annotation was reached (outer white annotation). Next, pMSCs were quantified using the HALO HighPlex FL package (**k**). A magnified micrograph of (**k**) is shown in (**l**) (grey box shows magnified area), demonstrating that pMSCs appear to be wrapped around the islet and display an elongated spindle-shaped morphology. Micrographs used in the representative figure were adjusted to optimise contrast and visibility without altering the underlying image data or quantification. Adjustments were made to the ‘Black In’, ‘White In’ and ‘Gamma’ settings. Donor ID: individual ≥13 years at type 1 diabetes diagnosis, 6563, nPOD

pMSCs, defined as immunopositive for CD90, CD73 and CD105 (Fig. 2a–d) and immunonegative for CD31, CD34 and CD45 (Fig. 2e–h), that were associated (either inside or within 10 μm of the outside of the islet) with islets (Fig. 2i, j) were identified (Fig. 2a–k, ESM Fig. 6). Across all 38 donors, 3796 pMSCs (7% of total pancreas MSCs) were associated with islets. A total of 1699 pMSCs at the islet periphery (within 10 µm of the outside of the islet) and 2097 intraislet pMSCs were identified. pMSCs appear to be wrapped around the islet periphery and display an elongated, spindle-shaped morphology, as expected (Fig. 2l).

Islets that had an associated pMSC were defined as islets with one or more pMSCs either within 10 µm of the islet periphery or within the islet itself (intraislet). The percentage of INS^+^GLU^+^ islets that had one or more associated pMSCs was higher in individuals with type 1 diabetes (≥39.5%) either <13 years or ≥13 years at diagnosis, compared with respective individuals of similar age without diabetes (≤20.3%; ESM Fig. 7).

### pMSC number and density are increased at the islet periphery of insulin-containing islets in individuals with type 1 diabetes

**Fig. 3:**
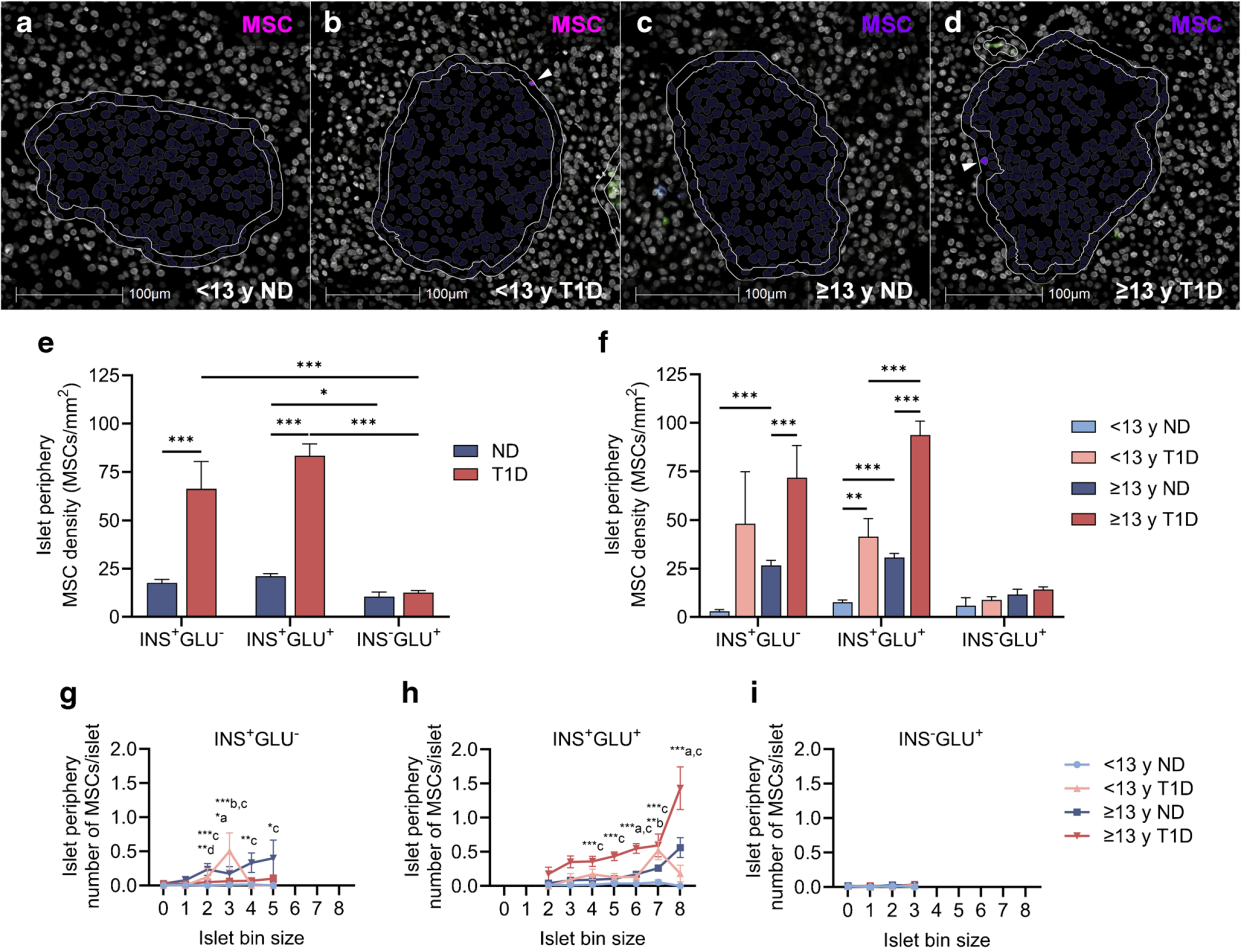
The density and number of pMSCs within a 10 µm region at the periphery of islets comprising different endocrine cell compositions and size. Representative quantifications of pMSCs (HighPlex FL package, HALO) at the islet periphery are shown in (**a**–**d**) (pMSCs highlighted by white arrows). For (**e**, **f**), data were derived from *n*=26,376 individual islets from 38 individuals (*n*=8 individuals <13 years at type 1 diabetes diagnosis and *n*=11 individuals ≥13 years at type 1 diabetes diagnosis [*n*=19 individuals with type 1 diabetes]; *n*=8 individuals <13 years without diabetes and *n*=11 individuals >13 years without diabetes [*n*=19 individuals without diabetes]). For (**g**–**i**), islet bin size containing <5 islets or bin sizes devoid of data for one or more groups were excluded; data were derived from *n*=23,290 individual islets. Bars represent mean ± SEM. Two-way ordinary ANOVA with post hoc tests adjusted for multiple comparisons with Bonferroni correction. Post hoc tests in (**e**): islets of the same endocrine cell composition; diabetes or no diabetes across islets of different endocrine cell compositions. Post hoc tests in (**f**): type 1 diabetes group and individuals of similar age without diabetes; type 1 diabetes and age of diagnosis; individuals without diabetes. Comparisons in (**e**, **f**) are shown with a horizontal black line between the two compared groups. Post hoc comparisons within each islet bin size in (**g, h**): ^a^<13 years at type 1 diabetes diagnosis vs ≥13 years at type 1 diabetes diagnosis; ^b^<13 years at type 1 diabetes diagnosis vs <13 years without diabetes; ^c^≥13 years at type 1 diabetes diagnosis vs ≥13 years without diabetes; ^d^<13 years without diabetes vs ≥13 years without diabetes. ***p≤0.001; **p≤0.01; *p≤0.05. Donor IDs: nPOD 6407 (**a**); nPOD 6578 (**b**); nPOD 6333 (**c**); nPOD 6362 (**d**). <13 y ND, <13 years without diabetes; <13 y T1D, <13 years at type 1 diabetes diagnosis; ≥13 y ND, ≥13 years without diabetes; ≥13 y T1D, ≥13 years at type 1 diabetes diagnosis; ND, no diabetes; T1D, type 1 diabetes

The number and density of pMSCs within a 10 µm region immediately outside the islet (islet periphery) were quantified (Fig. 3a–d). The density of pMSCs within 10 µm of insulin-containing (INS^+^GLU^−^ and INS^+^GLU^+^) but not insulin-deficient (INS^−^GLU^+^) islets was higher in individuals with type 1 diabetes compared with individuals without diabetes (*p*<0.001 for INS^+^GLU^−^ and INS^+^GLU^+^ islets, Fig. 3e). Accordingly, in type 1 diabetes the density of pMSCs at the periphery of insulin-deficient islets was dramatically reduced compared with islets containing residual beta cells (*p*<0.001), consistent with an islet-protective role for pMSCs. The density of pMSCs at the periphery of INS^+^GLU^+^ islets was higher in individuals who were ≥13 years at diagnosis of type 1 diabetes compared with those <13 years at diagnosis (*p*<0.001, Fig. 3f). When separated by islet size (Fig. 3g, h), the more pronounced increase in pMSC number at the periphery of islets in individuals ≥13 years compared with <13 years at diagnosis of diabetes was true for larger islets containing ~64–128 or ~265–512 cells (islet bin size 6 or 8; Fig. 3h).

In individuals without type 1 diabetes, pMSC density was also higher at the periphery of insulin-containing islets in individuals ≥13 years compared with individuals <13 years (Fig. 3f). Nonetheless, the density of pMSCs at the periphery of insulin-containing islets was increased to a greater extent in individuals with type 1 diabetes irrespective of the age at diagnosis when compared with individuals of similar age without diabetes (Fig. 3f). Irrespective of age or type 1 diabetes diagnosis, the mean number of pMSCs at the periphery of islets increased with islet size for insulin-containing (two-way ANOVA main effect islet bin size, *p*<0.001 for both) but not insulin-deficient (two-way ANOVA main effect islet bin size, *p*=0.61) islets (Fig. 3g–i). Thus, the number of pMSCs at the islet periphery increased with increasing numbers of beta cells.

### Intraislet pMSC density is increased within insulin-containing islets in individuals with type 1 diabetes

**Fig. 4:**
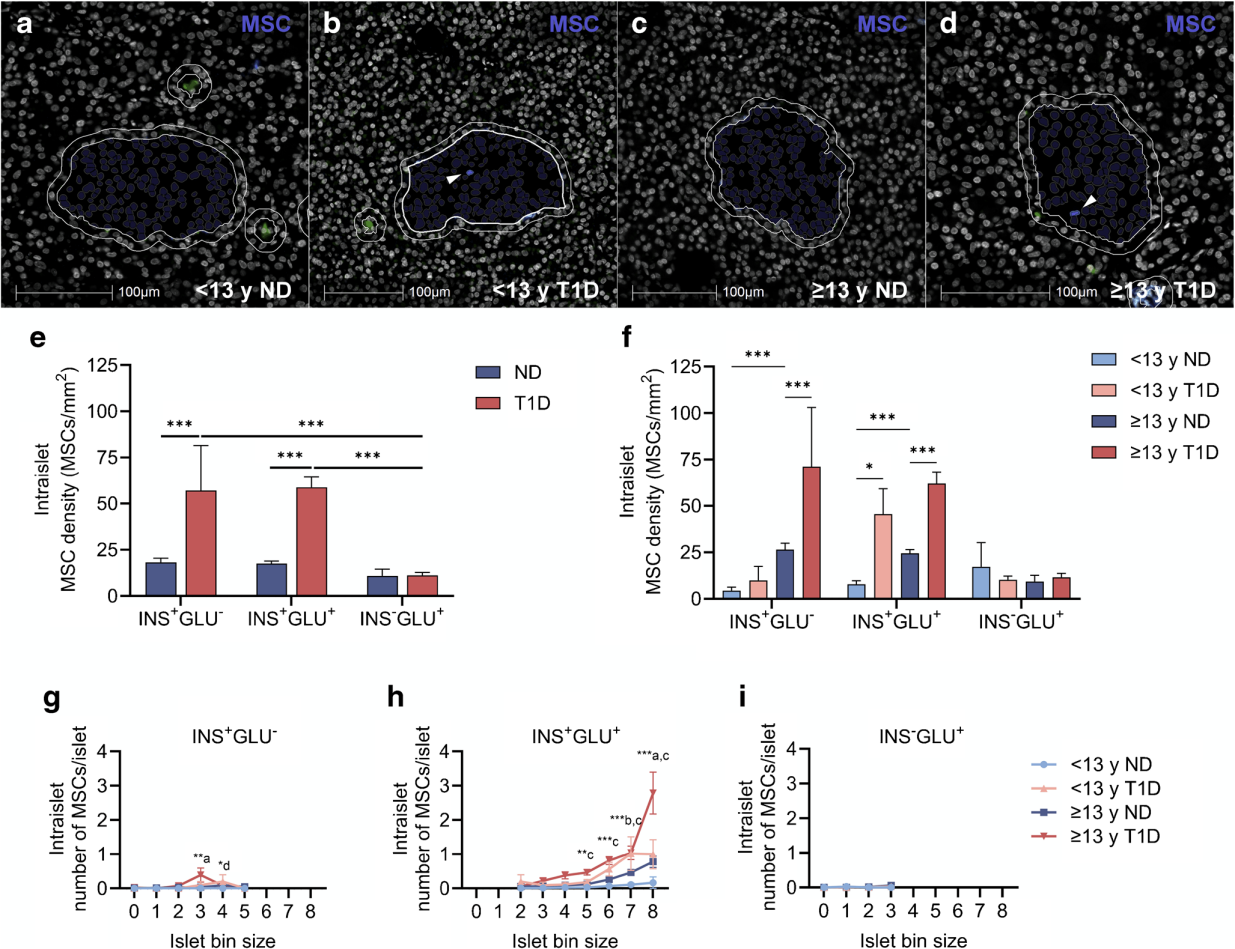
The density and number of intraislet pMSCs of islets comprising different endocrine cell compositions and size. Intraislet pMSCs (shown by white arrows, **a**–**d**) within INS^+^GLU^+^ islets are shown. Immunostaining for insulin (blue) and glucagon (green) outside of the analysed islets in (**a**–**d**) is shown. For (**e**, **f**), data were derived from *n*=26,376 individual islets from 38 individuals (*n*=8 individuals <13 years at type 1 diabetes diagnosis and *n*=11 individuals ≥13 years at type 1 diabetes diagnosis [*n*=19 individuals with type 1 diabetes]; *n*=8 individuals <13 years without diabetes and *n*=11 individuals >13 years without diabetes [*n*=19 individuals without diabetes]). For (**g–i**), islet bin size containing <5 islets or bin sizes devoid of data for one or more groups were excluded; data were derived from *n*=23,290 individual islets. Bars represent mean ± SEM. Two-way ordinary ANOVA with post hoc tests adjusted for multiple comparisons with Bonferroni correction. Post hoc tests in (**e**): islets of the same endocrine cell composition; diabetes or no diabetes across islets of different endocrine cell compositions. Post hoc tests in (**f**): type 1 diabetes group and individuals of similar age without diabetes; type 1 diabetes and age of onset; individuals without diabetes. Comparisons in (**e**, **f**) are shown with a horizontal black line between the two compared groups. Post hoc comparisons within each islet bin size in (**g**–**i**): ^a^<13 years at type 1 diabetes diagnosis vs ≥13 years at type 1 diabetes diagnosis; ^b^<13 years at type 1 diabetes diagnosis vs <13 years without diabetes; ^c^≥13 years at type 1 diabetes diagnosis vs ≥13 years without diabetes; ^d^<13 years without diabetes vs ≥13 years without. ***p≤0.001; **p≤0.01; *p≤0.05. Donor IDs: nPOD 6407 (**a**); nPOD 6578 (**b**); nPOD 6333 (**c**); nPOD 6362 (**d**). <13 y ND, <13 years without diabetes; <13 y T1D, <13 years at type 1 diabetes diagnosis; ≥13 y ND, ≥13 years without diabetes; ≥13 y T1D, ≥13 years at type 1 diabetes diagnosis; ND, no diabetes; T1D, type 1 diabetes

In accordance with our quantification of pMSCs at the islet periphery (Fig. 3a–f), the density of intraislet pMSCs within insulin-containing but not insulin-deficient islets was higher in individuals with type 1 diabetes compared with individuals without diabetes (*p*<0.001 for INS^+^GLU^−^ and INS^+^GLU^+^ islets, Fig. 4a–f). However, there did not appear to be a more pronounced intraislet pMSC density in individuals ≥13 years at diagnosis of type 1 diabetes compared with <13 years at diagnosis when all islet sizes were grouped together. When separated by islet size (Fig. 4g–i), only the largest INS^+^GLU^+^ islets showed increased intraislet pMSC number in individuals ≥13 years compared with <13 years at diagnosis of type 1 diabetes. In agreement with our observations for pMSC number at the islet periphery, the mean number of intraislet pMSCs increased with islet size for insulin-containing islets (two-way ANOVA main effect islet bin size, *p*<0.001 for both).

### pMSCs express islet-protective factors including ANXA1

**Fig. 5:**
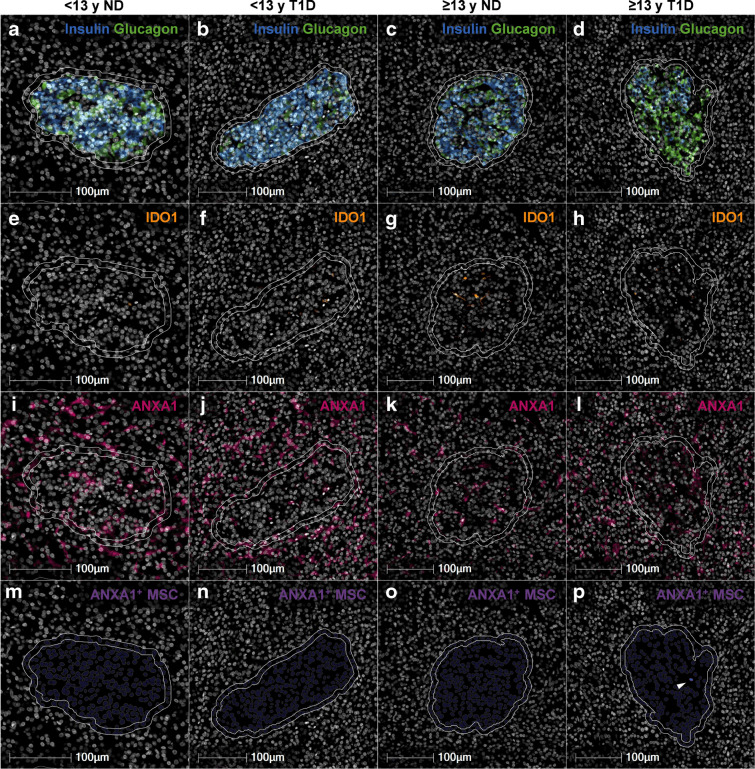
IDO1 and ANXA1 immunostaining and quantification of ANXA1^+^ pMSCs. Representative INS^+^GLU^+^ islets are shown. Islets were identified by insulin and glucagon immunostaining (**a**–**d**). IDO1 (**e**–**h**) and ANXA1 (**i**–**l**) immunostaining was conducted and ANXA1^+^ MSCs were quantified using the HALO HighPlex FL package (pMSCs shown by white arrows, **m**–**p**). Each micrograph (**a**–**p**) shows the islet annotation (inner white annotation) and expanded annotation 10 µm from the islet periphery (outer white annotation). Donor IDs: nPOD 6407 (**a**); nPOD 6578 (**b**); nPOD 6333 (**c**); nPOD 6362 (**d**). <13 y ND, <13 years without diabetes; <13 y T1D, <13 years at type 1 diabetes diagnosis; ≥13 y ND, ≥13 years without diabetes; ≥13 y T1D, ≥13 years at type 1 diabetes diagnosis

Isolated ‘exogenous’ MSCs secrete an array of cytoprotective, immunomodulatory and regenerative molecules in response to specific cues within their microenvironment to influence both islet cells and a wide range of immune cell subsets. In the current report we have determined whether the previously defined islet-protective and immunomodulatory factors, ANXA1 and IDO1, are expressed in human MSCs in vitro (ESM Fig. 8) and in pMSCs in the pancreas of individuals with and without type 1 diabetes (Fig. 5). The expression of *IDO1* was induced in human MSCs in vitro when exposed to cytokine combinations reflective of type 1 diabetes (IFN-γ + IL-1β [C_t_<18] and IFN-γ + IL-1β + TNF-α [C_t_<17]) compared with the no cytokine control (C_t_>30). *ANXA1* expression remained constitutively high across all groups (C_t_<21; ESM Fig. 8).

A total of 327 IDO1^+^ MSCs were identified across all pancreas sections (total pancreas including acinar, islets, ducts and vessels). The percentage of pMSCs that expressed IDO1 across all 38 individuals with and without type 1 diabetes was 1.02±0.34%. The percentage of pMSCs that were IDO1^+^ was similar for individuals with or without type 1 diabetes, irrespective of age [<13 years at type 1 diabetes diagnosis: 2.06±1.22%; <13 years without diabetes: 0.84±0.54%; ≥13 years at type 1 diabetes diagnosis: 1.15±0.64%; ≥13 years without diabetes: 0.25±0.13%; one-way ANOVA, *F*(3, 34)=1.156, *p*=0.32]. The small number of pMSCs expressing IDO1 indicates that IDO1 is not the primary islet-protective mechanism employed by pMSCs. Further analysis was not conducted on IDO1^+^ pMSCs due to the low number of cells detected.

In contrast, a total of 16,825 ANXA1^+^ pMSCs were identified across all pancreas sections. The percentage of total pancreas pMSCs that expressed ANXA1 across all 38 individuals with and without type 1 diabetes was 33.2% and was similar across groups, irrespective of age or type 1 diabetes diagnosis [<13 years at type 1 diabetes diagnosis: 35.78±6.20%; <13 years without diabetes: 27.38±5.08%; ≥13 years at type 1 diabetes diagnosis: 35.67±5.80%; ≥13 years without diabetes: 33.02±5.32%; one-way ANOVA, *F*(3, 34)=0.1867, *p*=0.73]. Of the total pancreas ANXA1^+^ pMSCs identified, 7.45% were associated with islets. Specifically, we detected 663 (3.94%) ANXA1^+^ intraislet pMSCs and 590 (3.51%) ANXA1^+^ pMSCs at the islet periphery (within 10 μm).

### The number and density of pMSCs expressing islet-protective ANXA1 at the islet periphery are higher in individuals with type 1 diabetes

**Fig. 6:**
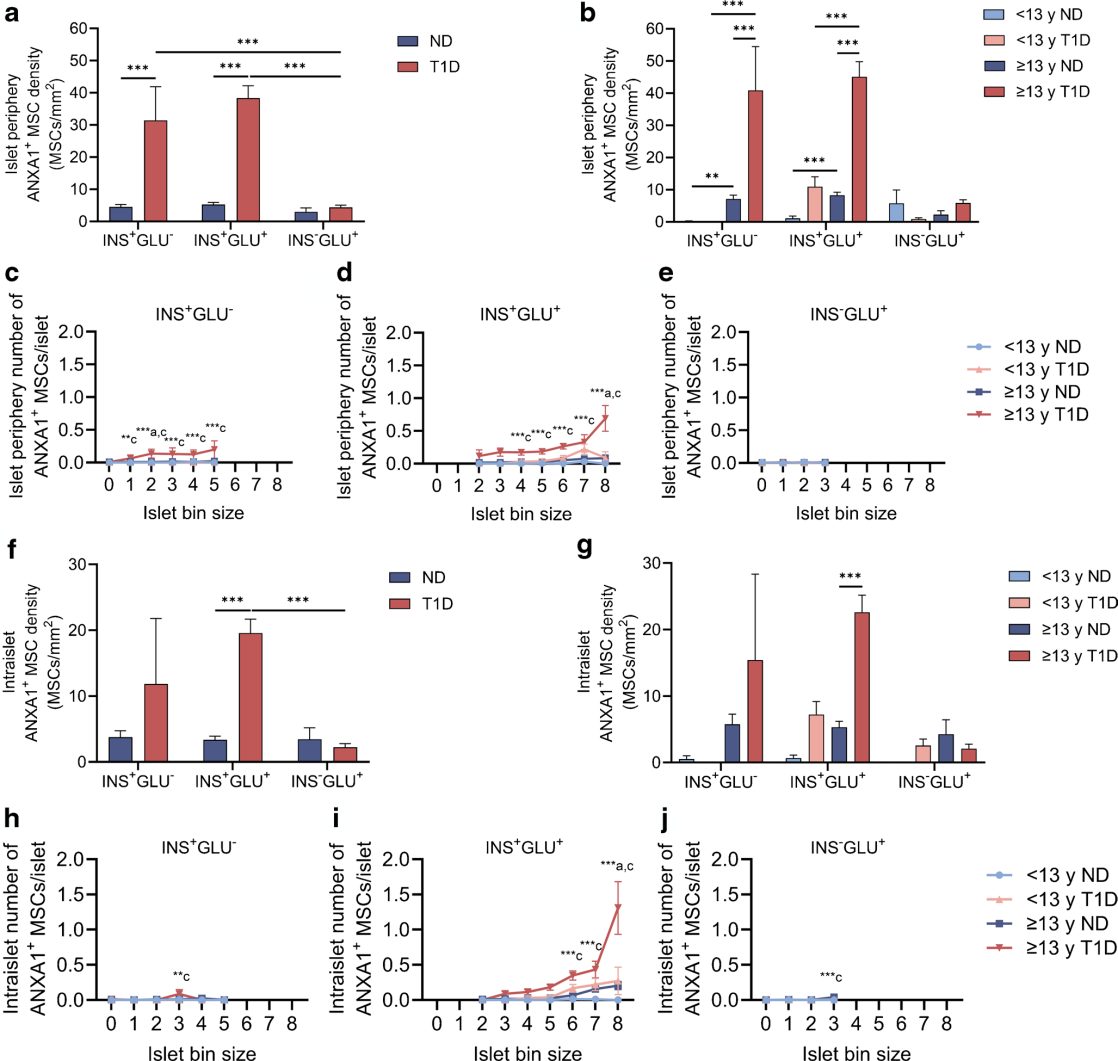
Number and density of pMSCs expressing islet-protective ANXA1 that are associated with islets. (**a**, **b**) and (**f**, **g**): data were derived from 26,376 individual islets from 38 individuals (*n*=8 individuals <13 years at type 1 diabetes diagnosis and *n*=11 individuals ≥13 years at type 1 diabetes diagnosis [*n*=19 individuals with type 1 diabetes]; *n*=8 individuals <13 years without diabetes and *n*=11 individuals >13 years without diabetes [*n*=19 individuals without diabetes]). (**c**–**e**) and (**h**–**j**): islet bin size containing <5 islets or bin sizes devoid of data for one or more groups were excluded; data were derived from *n*=23,290 individual islets. Bars represent mean ± SEM. Two-way ordinary ANOVA with post hoc tests adjusted for multiple comparisons with Bonferroni correction. Post hoc tests in (**a**) and (**f**): islets of the same endocrine cell composition; diabetes or no diabetes across islets of different endocrine cell compositions. Post hoc tests in (**b**) and (**g**): type 1 diabetes and individuals of similar age without diabetes; type 1 diabetes age of diagnosis; individuals without diabetes. Comparisons in (**a**, **b**) and (**f**, **g**) are shown with a horizontal black line between the two compared groups. Post hoc comparisons within each islet bin size in (**c**–**e**) and (**h**–**j**): ^a^<13 years at type 1 diabetes diagnosis vs ≥13 years at type 1 diabetes diagnosis; ^c^≥13 years at type 1 diabetes diagnosis vs ≥13 years without diabetes. ***p≤0.001; **p≤0.01. <13 y ND, <13 years without diabetes; <13 y T1D, <13 years at type 1 diabetes diagnosis; ≥13 y ND, ≥13 years without diabetes; ≥13 y T1D, ≥13 years at type 1 diabetes diagnosis; ND, no diabetes; T1D, type 1 diabetes

For individuals with type 1 diabetes, the density of ANXA1^+^ pMSCs was increased at the periphery of insulin-containing but not insulin-deficient islets (Fig. 6a). When segregated according to age at type 1 diabetes diagnosis, the density of ANXA1^+^ pMSCs was enhanced at the periphery (within 10 µm) of INS^+^GLU^+^ islets for individuals ≥13 years compared with individuals <13 years at diagnosis (Fig. 6b). When further separated according to islet size (Fig. 6c–e), enhanced ANXA1^+^ pMSC number at the islet periphery of INS^+^GLU^+^ islets was apparent for the largest islets (islet bin size 8) containing over ~256 cells (Fig. 6d). The number of ANXA1^+^ pMSCs at the islet periphery increased with islet size for insulin-containing islets (two-way ANOVA main effect islet bin size, *p*≤0.002 for both). In agreement with total pMSCs (both ANXA1^+^ and ANXA1^−^ pMSCs), this may suggest increased migration of ANXA1^+^ pMSCs to islets with an increasing number of beta cells.

The density and number of intraislet pMSCs that express ANXA1 (Fig. 6f–j) mostly aligned with the data for total (both ANXA1^+^ and ANXA1^−^ pMSCs) intraislet pMSCs within INS^+^GLU^+^ islets. An exception to this was that intraislet ANXA1^+^ pMSC density between individuals <13 years with and without type 1 diabetes was similar.

### Cell types expressing ANXA1 within the pancreas

Cell types including MSCs, immune cells, endothelial cells, ductal cells, pericytes and fibroblasts can express ANXA1. Our panel of MSC markers enabled us to investigate ANXA1^+^ MSCs, immune (CD45^+^) cells and endothelial (CD31^+^) cells. Overall, the density of ANXA1^+^ cells across the whole pancreas was increased in individuals with type 1 diabetes, compared with those without [1583±190 vs 899±81 ANXA1^+^ cells/mm^2^, respectively; *t*(36)=3.314, *p*=0.002].

The percentage of ANXA1^+^ cells that were MSCs (ANXA1^+^, CD73^+^, CD90^+^, CD105^+^, CD31^−^, CD34^−^, CD45^−^) was similar between individuals with and without type 1 diabetes [1.62±0.64% vs 1.76±0.77%, respectively; *t*(36)=0.1358, *p*=0.89].

The percentage of ANXA1^+^ cells that were immune cells (ANXA1^+^, CD45^+^) was increased in individuals with type 1 diabetes compared with those without [5.92±0.65% vs 3.73±0.71%, respectively; *t*(36)=2.282, *p*=0.03]. When separated by age, the percentage of ANXA1^+^ cells that were immune cells was similar between groups [<13 years at type 1 diabetes diagnosis: 5.49±0.45%; <13 years without diabetes: 3.40±1.09%; ≥13 years at type 1 diabetes diagnosis: 6.23±1.09%; ≥13 years without diabetes: 3.97±0.97%; one-way ANOVA, *F*(3, 34)=1.811, *p*=0.16].

The percentage of ANXA1^+^ cells that were endothelial cells (ANXA1^+^, CD31^+^) was also similar between individuals with and without type 1 diabetes [11.94±1.37% vs 8.82±1.01%, respectively; *t*(36)=1.832, *p*=0.08].

### Whole pancreas and individual-islet immune cell infiltration

Total pancreas CD45^+^ cell number was quantified as an index of inflammation. As expected, when categorised by age and diabetes status, whole pancreas inflammation was increased in individuals with type 1 diabetes compared with individuals without (two-way ANOVA main effect diabetes, *p*<0.001). When separated by age, irrespective of diabetes diagnosis, total pancreas inflammation was similar (*p*=0.76).

**Fig. 7:**
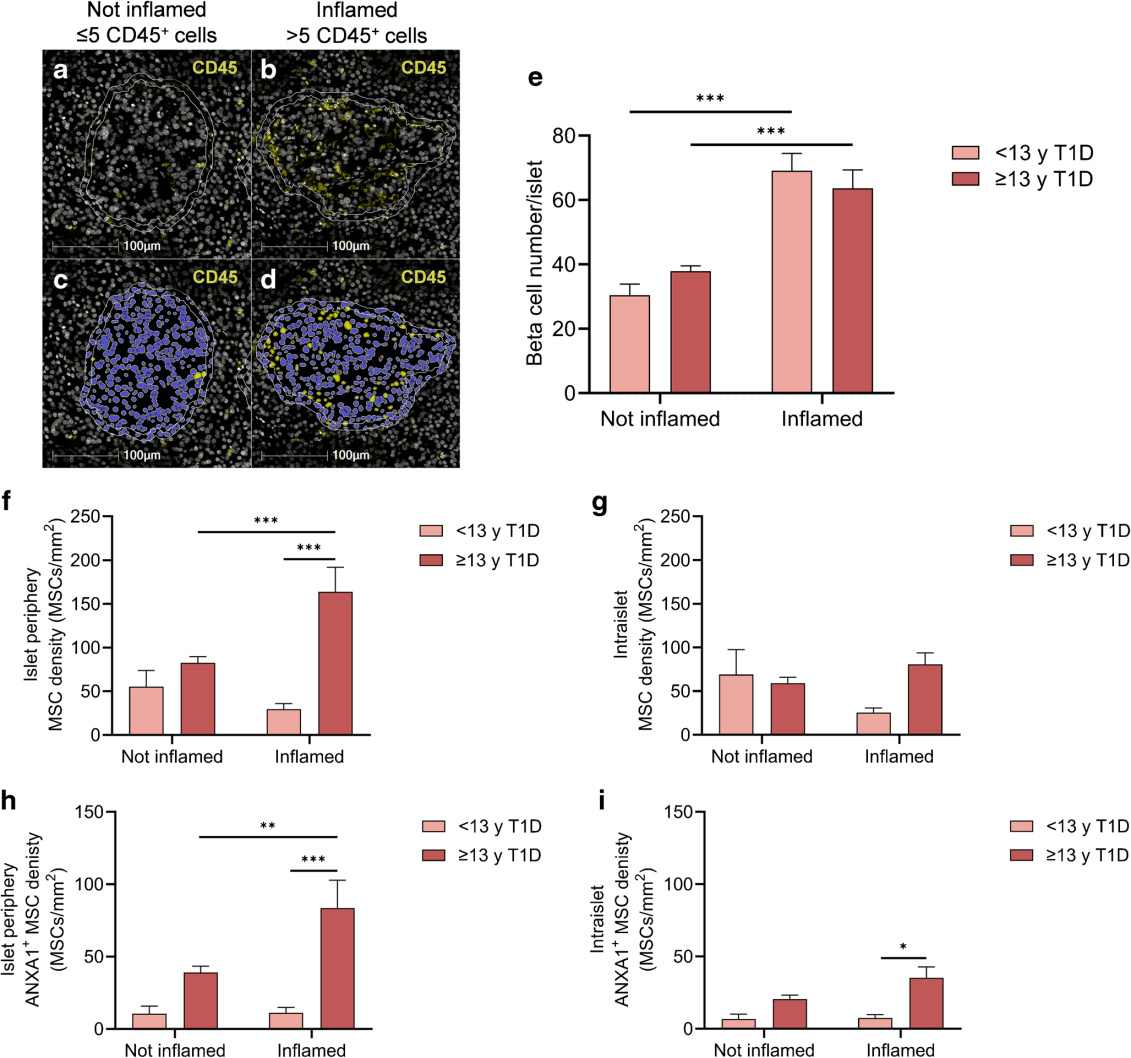
pMSCs are preferentially associated with inflamed islets in individuals older than 13 years at type 1 diabetes diagnosis. The number of CD45^+^ cells associated with islets (that were either within 10 µm of the islet periphery or inside the islet; **a**, **b**) were quantified, and islets were classified as not inflamed (≤5 CD45^+^ cells; **c**) or inflamed (>5 CD45^+^ cells; **d**). Islets are shown in (**a**–**d**) as inner white annotation and islet annotations expanded by 10 µm are shown by the outer annotation. (**e**–**i**) Data were derived from 1191 individual islets from *n*=8 individuals <13 years at type 1 diabetes diagnosis and *n*=11 individuals ≥13 years at type 1 diabetes diagnosis. Bars represent mean ± SEM. Two-way ordinary ANOVA with post hoc tests adjusted for multiple comparisons with Bonferroni correction. Post hoc tests in (**e**–**i**): between type 1 diabetes age of diagnosis at the same level of inflammation; between inflammation status for the same age of diagnosis. Comparisons are shown with a horizontal black line between the two compared groups. Donor ID: 6578, nPOD (**a**–**d**). ***p≤0.001; **p≤0.01; *p≤0.05. <13 y T1D, <13 years at type 1 diabetes diagnosis; ≥13 y T1D, ≥13 years at type 1 diabetes diagnosis

An islet was characterised as inflamed if there were more than five associated immune cells. This was determined by quantifying the total number of CD45^+^ cells located either inside the islet or within 10 µm of the islet periphery (Fig. 7a–d) [37, 38]. Very few (0.15%) INS^+^GLU^+^ islets from individuals without diabetes were categorised as inflamed, as expected [38]. Therefore, at the individual-islet level, inflammation was characterised further for all INS^+^GLU^+^ islets from individuals with type 1 diabetes only. The proportion of INS^+^GLU^+^ islets that were inflamed was 53.6% (*n*=126 islets) and 13.6% (*n*=130 islets) for individuals <13 years or ≥13 years at diagnosis of type 1 diabetes, respectively. The mean number of immune cells associated with each inflamed islet was higher for individuals <13 years at diagnosis of type 1 diabetes, compared with ≥13 years at diagnosis of type 1 diabetes [mean ± SEM; 27.48±3.35 vs 15.84±1.82 CD45^+^ cells; unpaired *t* test; *t*(254)=3.078, *p*=0.002]. This is consistent with the increased insulitis and more aggressive destruction of beta cells reported to occur in individuals with T1DE1 compared with T1DE2 [29, 30]. Whilst the number of beta cells per INS^+^GLU^+^ islet was not different between age groups, inflamed islets contained more beta cells than islets not under obvious immune attack (Fig. 7e), suggesting that larger islets may be preferentially infiltrated by immune cells.

pMSC density (ANXA1^+^ and ANXA1^−^) at the periphery of, but not within (intraislet), inflamed INS^+^GLU^+^ islets was increased in individuals ≥13 years at diagnosis of type 1 diabetes compared with islets that were not inflamed from the same group and compared with inflamed islets in individuals <13 years at diagnosis (Fig. 7f, g). The density of pMSCs that express ANXA1 at the periphery of inflamed INS^+^GLU^+^ islets displayed similar results to those of total pMSCs (ANXA1^+^ and ANXA1^−^; Fig. 7h). Intraislet ANXA1^+^ pMSC density was increased in inflamed INS^+^GLU^+^ islets of individuals ≥13 years compared with <13 years at type 1 diabetes diagnosis (Fig. 7i).

### Viability and proliferation of cytokine-exposed human MSCs in vitro

**Fig. 8:**
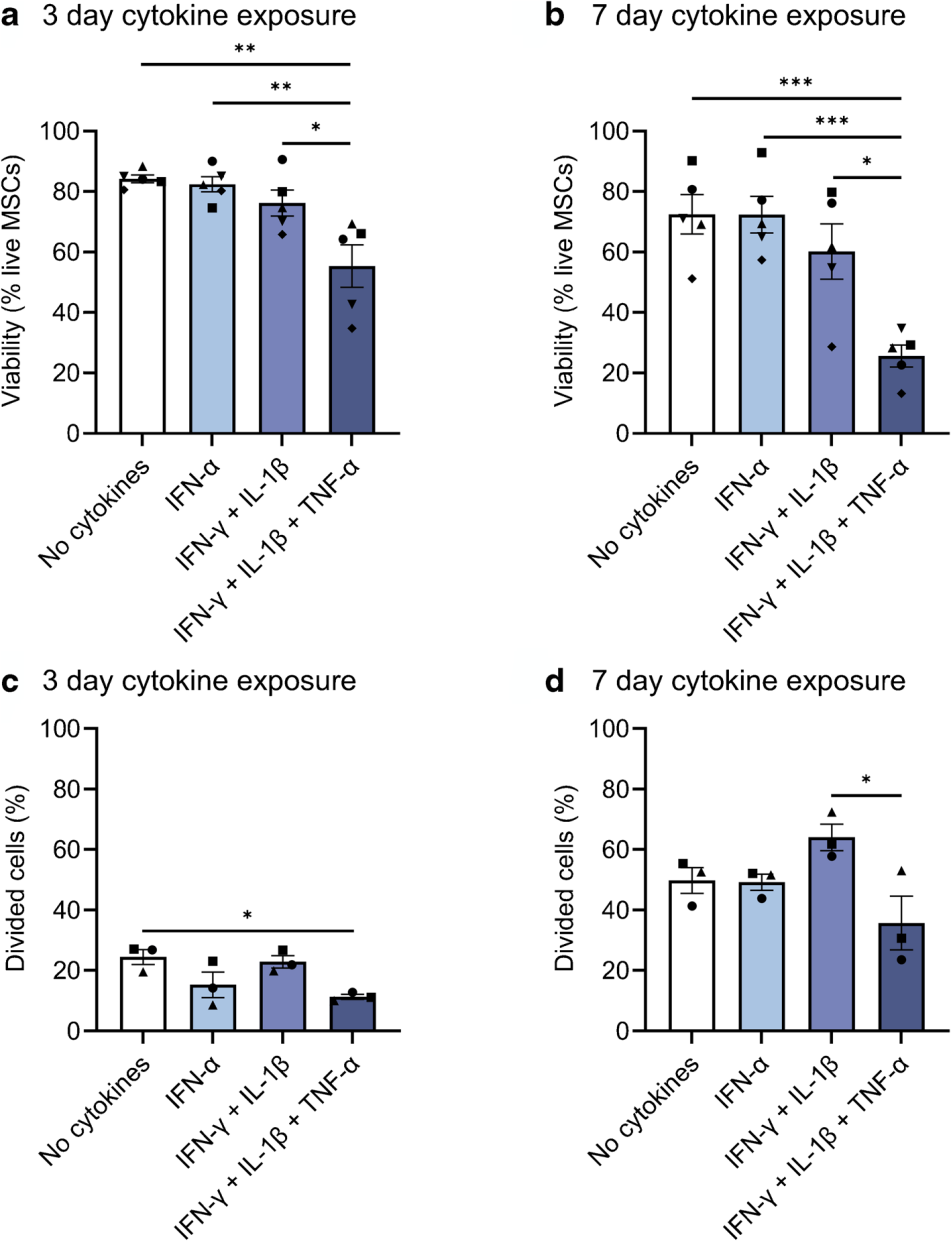
Viability and proliferation of cytokine-exposed human MSCs in vitro. Human MSCs were exposed to a cytokine condition of increasing aggression and viability was assessed after 3 (**a**) or 7 (**b**) days. The percentage of divided cells, as calculated by the FlowJo proliferation algorithm, was used to determine proliferation of CellTrace-stained cells after 3 (**c**) or 7 (**d**) days. Each symbol represents an independent experiment (*n*=5 for viability and *n*=3 for proliferation). Bars represent mean ± SEM. Human MSCs between passages 5 and 8 were used for all experiments. One-way ANOVA with post hoc tests adjusted for multiple comparisons with Bonferroni corrections. Comparisons in (**a**–**d**) are shown with a horizontal black line between the two compared groups. ***p≤0.001; **p≤0.01; *p≤0.05

We investigated the ability of MSCs to survive and proliferate under cytokine exposures of increasing ‘aggression’ and duration (Fig. 8a–d). MSC viability was reduced in response to the more aggressive cytokine cocktail (IFN-γ + IL-1β + TNF-α), compared with exposure to less aggressive conditions (IFN-α alone or IFN-γ + IL-1β) or to the no cytokine control following 3 or 7 day exposure (Fig. 8a, b). The percentage of MSCs that had divided over the course of the 3 day experiment was also reduced in response to the more aggressive cytokine cocktail compared with the no cytokine control (Fig. 8c).

Overall, exposure of MSCs to an aggressive cytokine combination led to increased cell death and reduced proliferation. This is particularly reflective of the cytokine exposure expected around islets in individuals <13 years at diagnosis of type 1 diabetes, where immune cell infiltration is more intense [29, 30]. This may indicate that pMSCs are less able to survive in situ at the islet periphery in individuals <13 years at diagnosis of type 1 diabetes.

## Discussion

To our knowledge, this is the first report providing a comprehensive immunohistological characterisation of pMSCs in individuals with and without type 1 diabetes. Using human pancreas tissue sections from both the nPOD and EADB collections, we have been able to monitor pMSC number, density and islet-protective phenotype close to diabetes onset (within 2 years) and to explore their association with inflamed islets in individuals with type 1 diabetes.

The current study introduces a methodological pipeline for phenotyping pMSCs on an individual-islet basis across whole-slide scans using the quantitative digital pathology platform HALO. This pipeline provides a reproducible framework for analysing cell phenotypes in individual regions of interest in a high-throughput manner across entire tissue sections. This pipeline can be adapted to identify other cell phenotypes across a range of tissue types, highlighting its applicability to different biological and disease contexts.

In vivo, native MSCs characteristically display similar phenotypic features to those reported in isolated (‘exogenous’), culture-expanded MSCs. MSCs have markers in common with other microvascular and stromal cell types including, but not limited to, endothelial cells [39], pericytes [24, 40, 41] and stellate cells [22, 42, 43]. As such, no single phenotypic marker distinguishes MSCs from other cell types. Thus, we have used multiplex fluorescent immunohistochemistry, using Opal 6-colour methodology, incorporating a comprehensive panel of human MSC markers (CD73^+^, CD90^+^, CD105^+^, CD31^−^, CD34^−^, CD45^−^) to unequivocally define MSC populations in human pancreas tissue from individuals with and without type 1 diabetes.

Automated quantitative image analysis following whole-slide scanning and multispectral unmixing has facilitated high-throughput analysis of 53,375 pMSCs across 38 individuals with and without type 1 diabetes. We have identified MSC populations in the exocrine tissue as well as intraislet MSCs, in accordance with reports that MSCs can be isolated from pancreatic ductal epithelial cells [44], acinar and endocrine pancreas tissue [20–26, 45, 46]. Moreover, we have demonstrated significant alterations in pMSC number and density associated with islets in individuals with type 1 diabetes.

MSCs distributed across the exocrine and endocrine pancreas are likely to contribute to tissue and immune system homeostasis in health (reviewed in [47]). MSCs respond to specific cues within their microenvironment and migrate to sites of injury, including pancreatic islets [19], where they exert their immunomodulatory and regenerative functions. Our detailed histological studies of the human pancreas have demonstrated that the number and density of exocrine pMSCs within the immediate vicinity (10 µm) of islets (specifically those still containing beta cells) are increased in individuals with type 1 diabetes. Furthermore, the percentage of INS^+^GLU^+^ islets that have pMSCs localised to the islet periphery was also markedly increased in type 1 diabetes compared with individuals without type 1 diabetes. Together with our finding that total pancreas MSC density was similar between individuals with and without type 1 diabetes, this indicates that the spatial distribution of exocrine pMSCs must also be altered in type 1 diabetes. Our analysis has intentionally focused on pancreas samples from individuals with recent-onset type 1 diabetes (≤2 years), where islet inflammation and immune attack are still ongoing [38]. This has enabled us to further demonstrate that pMSCs are preferentially associated with inflamed islets (more than five CD45^+^ immune cells either within the islet or within 10 µm of the islet periphery), indicating the selective homing of MSCs to inflamed human pancreatic islets.

We have also demonstrated that, in type 1 diabetes, pMSCs are preferentially localised to larger islets (bin size ≥6) containing a higher number of residual beta cells. This is perhaps not surprising given the established homing capacity of MSCs [19] towards islet-expressed chemokines including C-X-C motif chemokine ligand 12 (CXCL12) and C-X3-C motif ligand 1 (CX3CL1) [19, 48]. Thus, it is expected that islets with a higher number of residual beta cells would synthesise and release more MSC chemoattractants, generating a stronger chemokine gradient for MSC migration. This aligns with our hypothesis that MSCs support and protect islets, as larger insulin-containing islets with a higher MSC density are retained in type 1 diabetes.

Whilst our data support the notion that MSCs home to inflamed islets in the human pancreas during the progression of type 1 diabetes, it is beyond the scope of this study to definitively define the tissue origin of ‘pMSCs’ identified in the pancreas. Thus, although, we have standardised our terminology to pMSC for all MSC populations detected in the exocrine and endocrine pancreas, we acknowledge that pMSCs may well represent a population of ‘homing’ MSCs that originated from other tissue sources, such as bone marrow, as previously reported [19]. It is also plausible that there are subpopulations of pMSCS that have been generated through epithelial-to-mesenchymal transition from acinar or pancreatic ductal cells [49, 50].

One compelling mechanism to explain the increased number of pMSCs in type 1 diabetes is that subpopulation(s) of MSCs within the exocrine and/or endocrine pancreas arise due to the differentiation of pericytes into MSCs/pericyte-MSCs [47, 51]. Pericytes wrap around endothelial cells, behave as stem cells and can give rise to progenitor cells and MSCs when removed from the body and expanded ex vivo [51]. This process may reflect changes that occur in vivo under inflammatory conditions, such as those seen during type 1 diabetes pathogenesis. Pericytes are known to become myofibroblasts in fibrotic diseases, including islet vascular fibrosis [52], and to form native MSCs/pericyte-MSCs to mediate tissue repair. Indeed, proinflammatory cytokines influence pericytes to differentiate into native MSCs by loosening their physical association with endothelial cells and inducing their proliferation, migration and secretion of immunomodulatory factors [51, 53]. Whilst islet pericyte density has been reported to remain unchanged in type 1 diabetes, islet pericyte capillary coverage is reduced [54]. This may suggest that pericytes detach from the vasculature and differentiate into reparative pMSCs in type 1 diabetes. This hypothesis is supported by the current study which demonstrates an increased density of pMSCs associated with insulin-containing islets in individuals with type 1 diabetes.

Our studies consistently demonstrate that the increased number of pMSCs in individuals with type 1 diabetes is more pronounced in individuals with an older age at diagnosis (≥13 years). This could be explained by differences in the migratory capacity of MSCs with age. However, previous studies have demonstrated that MSC migration is reduced rather than enhanced with increasing age [55]. Alternatively, residual beta cells in the pancreas of individuals with later-onset type 1 diabetes may be better able to attract MSCs due to differences in their chemokine expression profile. Our in vitro measurements of MSC viability following exposure to proinflammatory cytokines suggest that the differences in pMSC number observed in situ are at least partially attributable to their ability to survive differentially aggressive cytokine cocktails with varying age of diabetes onset. Thus, our in vitro observations indicate that the increased number of pMSCs in individuals diagnosed with type 1 diabetes after the age of 13 years is likely to be related to the ability of MSCs to survive the less aggressive immune cell infiltration and attack typically associated with later diagnosis (≥13 years) of type 1 diabetes [29, 30]. Accordingly, whilst we have shown that MSC viability in vitro is not influenced by a 7 day exposure to IFN-α alone or a dual combination of IFN-γ and IL-1β, our studies have consistently demonstrated a dramatic reduction in MSC viability following exposure to a more aggressive cocktail of IFN-γ, IL-1β and TNF-α.

In health, the density of pMSCs either within or in proximity to insulin-containing islets increased with age, albeit to a lesser extent than that seen with type 1 diabetes. Notably, whole pancreas (total pancreas area including acinar, islets, ducts and vessels) MSC and immune (CD45^+^) density remained similar across age groups, suggesting that the observed increase in pMSC density associated with islets is unlikely to reflect age-related increases in inflammation. This is consistent with the relatively young age of the donors included in this study (all donors ≤27 years), where age-dependent pancreas inflammation would still be minimal [38]. These differences may be explained by the expected increase in beta cell maturation and function across age groups which is suggested to occur over the first three decades of life [56–58]. Indeed, the proportion of beta cells expressing endoplasmic reticulum stress, insulin secretion or autophagy-related genes was reported to increase in healthy donors of 10–19 years compared with <10 years [58]. Therefore, it is plausible that insulin-containing islets from healthy individuals ≥13 years in the present study produced increased levels of chemokines related to insulin secretion and tissue repair and provided a greater stimulus for MSC migration than in individuals <13 years.

Previous studies have revealed therapeutic mechanisms by which MSCs isolated from various postnatal tissues (including bone marrow, adipose, kidney and pancreas) then subsequently ‘culture-expanded’ improve islet function and survival [12, 59–64], as well as immune cell activity [5, 65, 66]. MSCs secrete an array of cytoprotective, immunomodulatory and regenerative molecules [7, 67–69] in response to specific cues within their microenvironment to influence both islet cells [59, 60, 70] and a wide range of immune cell subsets.

ANXA1 is an MSC-secreted islet G protein-coupled receptor ligand and is a key modulator of MSC-mediated improvements in islet functional survival following cytokine exposure in vitro [59, 60, 70]. In the current study we have demonstrated that a significant proportion (>25% for individuals with or without type 1 diabetes) of pMSCs within the immediate vicinity of human pancreatic islets express ANXA1 protein. Whilst the proportion of pMSCs at the islet periphery immunopositive for ANXA1 was comparable for individuals with and without type 1 diabetes, the number and density of ANXA1-immunopositive pMSCs were increased in individuals with type 1 diabetes. This reflects the diabetes-related increase in total (irrespective of ANXA1 expression) pMSC number and density at the islet periphery. Thus, diabetes-related alterations in the number and density of ANXA1-immunopositive pMSCs do not appear to be due to the induction of ANXA1 expression in pMSCs exposed to diabetogenic stressors. Accordingly, we have demonstrated constitutively high expression of *ANXA1* in culture-expanded MSCs, both in the absence of cytokines [59, 60] as well as following exposure to differentially aggressive cytokine cocktails. The current observations indicate that a protective role of pMSCs in the human pancreas is likely to be at least in part attributable to the anti-inflammatory functions of ANXA1 [71, 72].

Immunosuppression by human MSCs is known to be mediated by IDO1, an enzyme that catalyses the rate-limiting step in the degradation of tryptophan, along the kynurenine pathway [73]. Thus, MSCs downregulate T cell proliferation and functioning, in part via IDO-mediated tryptophan catabolism [5, 73, 74]. We therefore sought to determine whether pMSCs in the human pancreas of individuals with recent-onset type 1 diabetes, where immune attack is ongoing, express IDO1. In contrast to the relatively high proportion of pMSCs (>25%) found to express the anti-inflammatory protein ANXA1, our study shows that less than 2% of pMSCs are immunopositive for IDO1.

Our in vitro studies demonstrate that MSC *IDO1* expression is minimal without cytokine stimulation and is induced following exposure to cytokine combinations of varying intensity, consistent with previous studies [74]. The low numbers of MSCs immunopositive for IDO1 in the human pancreas of individuals both with and without type 1 diabetes may highlight a potential defect in the ability of endogenous pMSCs to perform their IDO1-mediated immunosuppressive functions during type 1 diabetes progression. Alternatively, IDO1 may be only transiently expressed by pMSCs, thereby reducing the likelihood of detecting IDO1-positive pMSCs in situ but nonetheless enabling pMSCs to exert their established immunomodulatory effects on T cell function within the local pancreatic microenvironment.

MSCs have a plethora of anti-inflammatory, immunomodulatory and regenerative properties (reviewed in [65, 75]), including the functional capacity to reduce CD4^+^ and CD8^+^ T cell as well as B cell proliferation and activity. Our in situ studies of the human pancreas suggest that MSCs are recruited to islets undergoing immune attack where they express anti-inflammatory islet-protective factors, including ANXA1, to preserve endogenous islet beta cell survival. Accordingly, in type 1 diabetes the density of both total pMSCs and ANXA1^+^ pMSCs at the periphery of insulin-deficient islets was dramatically reduced compared with islets containing residual beta cells. Furthermore, the density and number of ANXA1^+^ pMSCs observed at the periphery of insulin-containing islets were higher in individuals with later-onset type 1 diabetes (≥13 years), corresponding to the less aggressive rate of type 1 diabetes progression known to occur in these individuals [29, 30]. Thus, our current study supports an islet-protective role for endogenous pMSCs in delaying type 1 diabetes progression.

Our conclusions are based on data generated from individuals of European descent. Future studies are required to confirm whether the number, distribution or immunomodulatory phenotype of pMSCs differs with ethnicity, which may be expected due to the heterogeneity of type 1 diabetes between ethnic groups (reviewed in [31]). We have shown that a comprehensive panel of six markers, aligned to the ISCT minimal criteria for defining human MSCs [27, 28], is required to accurately identify MSCs in situ. Thus, endocrine cell types and MSC-expressed islet-protective factors were identified on adjacent 4 µm sections and aligned in HALO to minimise distance-related effects. This methodology enabled resourceful use of finite pancreas tissue from the EADB and nPOD collections, maximising insights into the characterisation of MSCs in the human pancreas.

Our study intentionally focused on individuals with short-duration type 1 diabetes, where immune cell infiltration is still ongoing [38]. This enabled us to demonstrate that pMSCs were preferentially associated with inflamed islets in individuals ≥13 years at diagnosis of type 1 diabetes. Our future studies aim to further characterise immune cell subpopulations associated with inflamed islets and their relationship to pMSCs in individuals with short-duration type 1 diabetes. This will build upon our current observation demonstrating that a higher percentage of ANXA1^+^ cells express the pan-immune cell marker CD45 in individuals with type 1 diabetes. In addition, these studies will further investigate pMSCs and likely alterations in their distribution throughout the exocrine tissue. Future studies will also be directed towards investigating whether the increased pMSC density associated with individuals ≥13 years is maintained into longer-duration disease where beta cell loss is more pronounced, and islet inflammation is relatively resolved.

Altogether, the results presented here suggest a protective role for pMSCs in delaying the progression of type 1 diabetes. We suggest that pMSCs localised at the periphery of insulin-containing islets may play a protective role to limit CD8^+^ T cell-mediated destruction of islets. In accord with this, our detailed observations demonstrate that: (1) pMSCs are more abundant in the vicinity of insulin-containing islets when compared with insulin-deficient islets; (2) pMSCs are more abundant in individuals with older-onset type 1 diabetes (age at diagnosis ≥13 years), corresponding with a less aggressive immune cell infiltration; (3) pMSCs are preferentially associated with larger islets containing a greater number of residual beta cells; and (4) pMSCs express therapeutic factors, namely ANXA1, that have established roles in protecting islets from cytokine-induced apoptosis and preserving insulin secretory function [59, 60].

Therapeutic strategies to modulate the homing of endogenous or systemically infused exogenous MSCs to pancreatic islets represent a promising way to enhance the clinical applications of MSCs to preserve endogenous beta cells. In addition, enhancing the resilience of MSCs to aggressive cytokine exposure may improve their clinical application in individuals with younger-onset type 1 diabetes where MSC survival is challenged by the aggressive inflammatory pancreas environment. Overall, our data suggest that therapeutically administered MSCs are likely to be more effective at preserving endogenous beta cell mass in individuals with later-onset type 1 diabetes where immune cell infiltration is less aggressive and MSCs have a greater ability to survive and exert their reparative functions.

## Supplementary Information

Below is the link to the electronic supplementary material.

ESM (PDF 1888 KB)

## Abbreviations

ANXA1: Annexin A1
EADB: Exeter Archival Diabetes Biobank
HIER: Heat-induced epitope retrieval
IDO1: Indoleamine 2,3-dioxygenase 1
INS^+^GLU^−^: Insulin-positive glucagon-negative
INS^+^GLU^+^: Insulin-positive glucagon-positive
INS^−^GLU^+^: Insulin-negative glucagon-positive
ISCT: International Society for Cell and Gene Therapy
MSC: Mesenchymal stromal cell
nPOD: Network for Pancreatic Organ Donors with Diabetes
pMSC: Pancreatic mesenchymal stromal cell
T1DE1: Type 1 diabetes endotype 1
T1DE2: Type 1 diabetes endotype 2
