# Supplementary material for: Characterisation of human pancreatic mesenchymal stromal cells in type 1 diabetes
Source: Diabetologia. 2025 Dec 21;69(4):966–86. doi: 10.1007/s00125-025-06634-w (PMC12957432; doi:10.1007/s00125-025-06634-w)
Supplement: Supplementary file 1 — ESM (PDF 1888 KB) [file 125_2025_6634_MOESM1_ESM.pdf]

Electronic Supplementary Material

Characterisation of human pancreatic mesenchymal stromal cells in type 1 diabetes

Rebecca E. Dewhurst-Trigg, Jocelyn Atkins, Noel G. Morgan, Martin Eichmann, Sarah J. Richardson and Chloe L. Rackham

Exeter Centre for Excellence in Diabetes, Department of Clinical and Biomedical Sciences, University of Exeter, Exeter, UK.

Content:

ESM Tables

ESM Figures

ESM Tables

ESM Table 1 Clinical characteristics of pancreas donors included in this study from the EADB and nPOD collections.

Tissue collection	Donor ID RRID	Group	Age (years)	Sex	T1D duration (y)	BMI (kg/m^2)	C-Peptide (ng/ml)	HbA1C	Islet autoantibody positivity
EADB	E428 SAMN46311904	<13 y T1D	5	M	0.02	Unknown	Unknown	Unknown	Unknown
EADB	E375 SAMN46311885	<13 y T1D	11	F	0.02	Unknown	Unknown	Unknown	Unknown
EADB	SC115 SAMN46311955	<13 y T1D	1	F	0.01	Unknown	Unknown	Unknown	Unknown
EADB	485/88 SAMN46312114	<13 y ND	2	F	N/A	Unknown	Unknown	Unknown	Unknown
EADB	12426 SAMN46312017	<13 y ND	5	Unknown	N/A	Unknown	Unknown	Unknown	Unknown
EADB	315/89 SAMN46312086	<13 y ND	9	M	N/A	Unknown	Unknown	Unknown	Unknown
EADB	E386 SAMN46311889	≥13 y T1D	15	M	0.5	Unknown	Unknown	Unknown	Unknown
EADB	SC57 SAMN46311975	≥13 y T1D	18	F	0.02	Unknown	Unknown	Unknown	Unknown
EADB	SC76 SAMN46311979	≥13 y T1D	20	M	0.06	Unknown	Unknown	Unknown	Unknown
EADB	146/66 SAMN46312039	≥13 y ND	18	F	N/A	Unknown	Unknown	Unknown	Unknown
EADB	PAN8 SAMN46312163	≥13 y ND	19	Unknown	N/A	Unknown	Unknown	Unknown	Unknown
EADB	333/66 SAMN46312091	≥13 y ND	16	M	N/A	Unknown	Unknown	Unknown	Unknown

nPOD	6533 SAMN18242777	<13 y T1D	3.75	F	0	17.7	0.17	11.4	IA2A+ mIAA+ ZnT8A+
nPOD	6534 SAMN18242778	<13 y T1D	4.19	F	0	22.6	0.05	15.6	IA-2A+
nPOD	6578 SAMN33284295	<13 y T1D	11.95	F	0	22.5	0.35	13.6	IA2A+ ZnT8A+
nPOD	6209 SAMN15879265	<13 y T1D	5	F	0.25	15.9	0.1	Unknown	IA-2A+ ZnT8A+ mIAA+
nPOD	6371 SAMN15879424	<13 y T1D	12.5	F	2	16.6	0.11	9.5	GADA+ IA-2A+ mIAA+ ZnT8A+
nPOD	6407 SAMN15879460	<13 y ND	4.6	F	N/A	16	5.35	5.5	Negative
nPOD	6488 SAMN15879541	<13 y ND	4.6	F	N/A	16.8	8.67	5.6	Negative
nPOD	6382 SAMN15879435	<13 y ND	4.7	F	N/A	17.2	5.21	5.3	Negative
nPOD	6293 SAMN15879347	<13 y ND	9	F	N/A	18.6	2.22	Unknown	Negative
nPOD	6413 SAMN15879466	<13 y ND	10.1	F	N/A	19	5.27	5.6	Negative
nPOD	6228 SAMN15879284	≥13 y T1D	13	M	0	17.4	0.1	13.3	GADA+ IA-2A+ ZnT8A+
nPOD	6563 SAMN30386851	≥13 y T1D	14.56	F	0	25.5	1.04	9.6	IA2A+
nPOD	6551 SAMN25652262	≥13 y T1D	20.7	M	0.58	23.1	0.11	6.4	GADA+ IA-2A+ mIAA+ ZnT8A+
nPOD	6520 SAMN18053203	≥13 y T1D	21.61	M	0	29.3	0.37	11.9	GADA+ IA-2A+ ZnT8A+
nPOD	6362 SAMN15879415	≥13 y T1D	24.9	M	0	28.5	0.38	10	GADA+

nPOD	6550 SAMN25652261	≥13 y T1D	25.06	M	0	16.4	<0.02	14	GADA+ ZnT8A+
nPOD	6579 SAMN33284296	≥13 y T1D	13.91	F	1.167	18.4	0.31	15	GADA+ mIAA+
nPOD	6469 SAMN15879522	≥13 y T1D	26.06	F	1.5	26.9	0.66	7.4	GADA+
nPOD	6501 SAMN15879554	≥13 y ND	12.85	M	N/A	15.8	9.69	5.2	Negative
nPOD	6374 SAMN15879427	≥13 y ND	14	F	N/A	18.9	13.42	6.1	Negative
nPOD	6548 SAMN25652259	≥13 y ND	20.24	M	N/A	23.8	4.04	5.7	Negative
nPOD	6339 SAMN15879393	≥13 y ND	23.3	M	N/A	25	10.56	5.3	Negative
nPOD	6431 SAMN15879484	≥13 y ND	13.79	M	N/A	23.1	1.32	5.4	Negative
nPOD	6271 SAMN15879325	≥13 y ND	17	M	N/A	24.4	11.47	Unknown	Negative
nPOD	6232 SAMN15879288	≥13 y ND	14	F	N/A	20.83	19.5	Unknown	Negative
nPOD	6333 SAMN15879387	≥13 y ND	27.1	F	N/A	24.9	9.37	4.7	Negative

<13 y ND, <13 years without diabetes; ≥13 y ND, ≥13 years without diabetes; <13 y T1D, <13 years at type 1 diabetes diagnosis; ≥13 y T1D, ≥13 years at type 1 diabetes diagnosis.

ESM Table 2 Optimised Opal panel to identify pMSCs.

Antigen	Antibody (clone)<br>Product number<br>Dilution<br>Host species<br>RRID	HIER	HRP secondary	Opal fluorophore<br>Dilution	Position in staining panel
CD90	CD90 (EPR3132)<br>Ab92574; Abcam<br>1/100<br>Rb mAb<br>AB_10563647	Citrate<br>pH 6	Opal Ms/Rb HRP	570<br>1/50	1
CD105	CD105 (EPR10145-12)<br>Ab169545; Abcam<br>1/100<br>Rb mAb<br>AB_2894873	TE<br>pH 9	Opal Ms/Rb HRP	690<br>1/50	2
CD31	CD31 (EPR3094)<br>Ab76533; Abcam<br>1/80<br>Rb mAb<br>AB_1523298	TE<br>pH 9	Opal Ms/Rb HRP	480<br>1/50	3
CD45	CD45 (2B11 + PD7/26)<br>M0701; Agilent Dako<br>1/200<br>Ms mAb<br>AB_2314143	Citrate<br>pH 6	Opal Ms/Rb HRP	620<br>1/50	4
CD73	CD73 (D7F9A)<br>13160; Cell Signaling<br>1/100<br>Rb mAb<br>AB_2716625	Citrate<br>pH 6	Opal Ms/Rb HRP	520<br>1/50	5
CD34	CD34 (QBEnd 10)<br>MA110202; Invitrogen<br>1/300<br>Ms mAb<br>AB_11156010	TE<br>pH 9	Opal Ms/Rb HRP	780<br>1/25	6

Primary antibodies were diluted in antibody diluent (Agilent Dako) and Opal fluorophores were diluted in amplification diluent (Akoya Biosciences).
HRP, horseradish peroxidase; mAb, monoclonal antibody; Ms, mouse; Rb, rabbit; TE, tris EDTA.

ESM Table 3 Optimised Opal panel to identify islets and MSC-derived islet-protective factors.

Antigen	Antibody (clone)<br>Product number<br>Dilution<br>Host species<br>RRID	HIER	HRP secondary	Opal fluorophore<br>Dilution	Position in staining panel
CD90	CD90 (EPR3132)<br>Ab92574; Abcam<br>1/100<br>Rb mAb<br>AB_10563647	Citrate<br>pH 6	Opal Ms/Rb<br>HRP	570<br>1/50	1
IDO1	IDO1 (EPR20374)<br>Ab211017; Abcam<br>1/500<br>Rb mAb<br>AB_2936946	TE<br>pH 9	Opal Ms/Rb<br>HRP	480<br>1/75	2
ANXA1	ANXA1 (EPR19342)<br>Ab214486; Abcam<br>1/200<br>Rb mAb<br>AB_2890907	TE<br>pH 9	Opal Ms/Rb<br>HRP	520<br>1/75	3
CD45	CD45 (2B11 + PD7/26)<br>M0701; Agilent Dako<br>1/200<br>Ms mAb<br>AB_2314143	Citrate<br>pH 6	Opal Ms/Rb<br>HRP	620<br>1/50	4
Insulin	Insulin (ICBTACLS)<br>14-9769-82; Invitrogen<br>1/1500<br>Ms mAb<br>AB_2573014	Citrate<br>pH 6	Opal Ms/Rb<br>HRP	690<br>1/100	5
Glucagon	Glucagon (K79bB10)<br>Ab10988, Abcam<br>1/800<br>Ms mAb<br>AB_297642	Citrate<br>pH 6	Opal Ms/Rb<br>HRP	780<br>1/25	6

Primary antibodies were diluted in antibody diluent (Agilent Dako) and Opal fluorophores were diluted in amplification diluent (Akoya Biosciences).
HRP, horseradish peroxidase; mAb, monoclonal antibody; Ms, mouse; Rb, rabbit; TE, tris EDTA.

ESM Table 4 Qiagen QuantiTect quantitative PCR primer assays.

**Gene name**	**Primer assay**
ANXA1	QT00078197
IDO1	QT00000504
ACTB	QT00095431
GAPDH	QT00079247
HPRT1	QT00059066
PPIA	QT01866137

ESM Table 5 Number and percentage of islets comprising different endocrine cell compositions among individual donors with and without type 1 diabetes from the EADB and nPOD collections.

		**Absolute number of islets (*n*)**				**Percentage of total islets (%)**		
**Donor ID**	**Group**	**$INS^{+}GLU^{-}$**	**$INS^{+}GLU^{+}$**	**$INS^{-}GLU^{+}$**	**Total islets**	**$INS^{+}GLU^{-}$**	**$INS^{+}GLU^{+}$**	**$INS^{-}GLU^{+}$**
E428B	<13 y T1D	4	11	157	172	2.33	6.40	91.28
E375	<13 y T1D	0	6	83	89	0.00	6.74	93.26
SC115	<13 y T1D	2	9	193	204	0.98	4.41	94.61
6533	<13 y T1D	20	11	782	813	2.46	1.35	96.19
6534	<13 y T1D	4	43	184	231	1.73	18.61	79.65
6578	<13 y T1D	57	94	143	294	19.39	31.97	48.64
6209	<13 y T1D	3	39	532	574	0.52	6.79	92.68
6371	<13 y T1D	1	22	251	274	0.36	8.03	91.61
6407	<13 y ND	193	281	93	567	34.04	49.56	16.40
6488	<13 y ND	937	553	33	1523	61.52	36.31	2.17
6382	<13 y ND	443	403	47	893	49.61	45.13	5.26
6293	<13 y ND	106	549	76	731	14.50	75.10	10.40
6413	<13 y ND	247	304	28	579	42.66	52.50	4.84
12426	<13 y ND	360	498	20	878	41.00	56.72	2.28
31589	<13 y ND	116	96	2	214	54.21	44.86	0.93
48588	<13 y ND	470	575	22	1067	44.05	53.89	2.06
E386	≥13 y T1D	1	38	136	175	0.57	21.71	77.71
SC57	≥13 y T1D	3	12	7	22	13.64	54.55	31.82
SC76	≥13 y T1D	98	84	146	328	29.88	25.61	44.51
6228	≥13 y T1D	15	80	1242	1337	1.12	5.98	92.89
6563	≥13 y T1D	48	143	251	442	10.86	32.35	56.79
6551	≥13 y T1D	13	85	166	264	4.92	32.20	62.88
6520	≥13 y T1D	108	181	472	761	14.19	23.78	62.02
6362	≥13 y T1D	2	147	920	1069	0.19	13.75	86.06

6550	≥13 y T1D	7	91	179	277	2.53	32.85	64.62
6579	≥13 y T1D	2	24	510	536	0.37	4.48	95.15
6469	≥13 y T1D	5	71	1358	1434	0.35	4.95	94.70
6501	≥13 y ND	664	480	149	1293	51.35	37.12	11.52
6374	≥13 y ND	748	488	106	1342	55.74	36.36	7.90
6548	≥13 y ND	232	89	19	340	68.24	26.18	5.59
6339	≥13 y ND	488	642	157	1287	37.92	49.88	12.20
6431	≥13 y ND	461	444	129	1034	44.58	42.94	12.48
6271	≥13 y ND	1014	981	468	2463	41.17	39.83	19.00
6232	≥13 y ND	366	664	216	1246	29.37	53.29	17.34
6333	≥13 y ND	264	305	38	607	43.49	50.25	6.26
33366	≥13 y ND	108	129	14	251	43.03	51.39	5.58
14666	≥13 y ND	81	136	1	218	37.16	62.39	0.46
PAN8	≥13 y ND	321	185	41	547	58.68	33.82	7.50

<13 y ND, <13 years without diabetes; <13 y T1D, <13 years at type 1 diabetes diagnosis; ≥13 y ND, ≥13 years without diabetes; ≥13 y T1D, ≥13 years at type 1 diabetes diagnosis.

ESM Figures

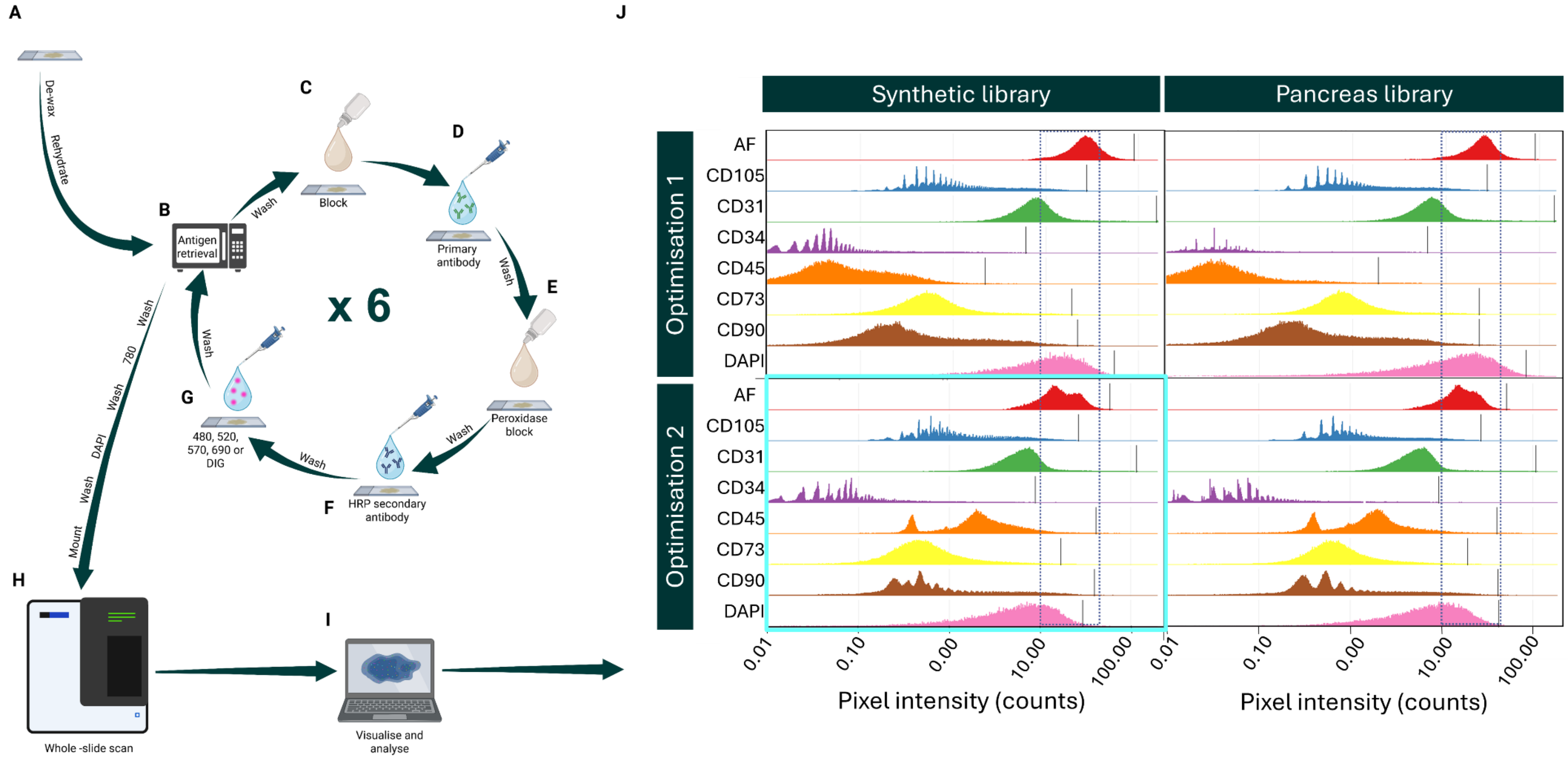

ESM Fig. 1 Optimisation of Opal-Tyramide signal amplification 6-plex immunohistochemistry to identify pMSCs.
Tissue sections (**A**) were subjected to 6 rounds of opal staining including heat-induced epitope retrieval (**B**), blocking (**C**), primary antibody incubation (**D**), endogenous peroxidase blocking (**E**), host-specific horseradish peroxidase-conjugated secondary antibody incubation (**F**), and signal generation (**G**). Whole-slide scans were acquired using the PhenoImager HT (Akoya Biosciences; **H**) and visualised (**I**).

Six-plex opal immunohistochemistry required optimisation (**J**) before a final protocol could be confirmed. Optimisation was first performed (optimisation 1) with staining conditions informed by Akoya database of antigen clone-specific positioning experiments and our previous experiments, in EADB pancreas and positive control tissue. Single-stained library and autofluorescence slides were also prepared with EADB pancreas tissue.
Images were unmixed using a custom pancreas library and the Akoya-provided synthetic library for comparison. PhenoptrReports Component Levels Report was performed in R to investigate staining intensity (pixel intensity) and signal balance between fluorophores. A second optimsation was performed to refine and balance fluorophore signal intensity. Vertical lines along the histogram for each antigen are representative of the 99.9% percentile pixel and are representative of positive signal. The dashed rectangle shows optimal pixel intensity.
There was little difference between the custom pancreas library and the Akoya synthetic library in optimisation 2. Therefore, in the interest of pancreas tissue preservation, the synthetic library was chosen. Light blue box highlighting Optimisation 2, Synthetic library data shows the methodology confirmed as the final protocol.
Donor IDs for tissue required for 6-plex opal and library/autofluorescence optimisation: PAN1, P39/67, E560, 8503, 6771/86, 88/66. Data shown in this figure from 88/66.
Figure creating using BioRender.

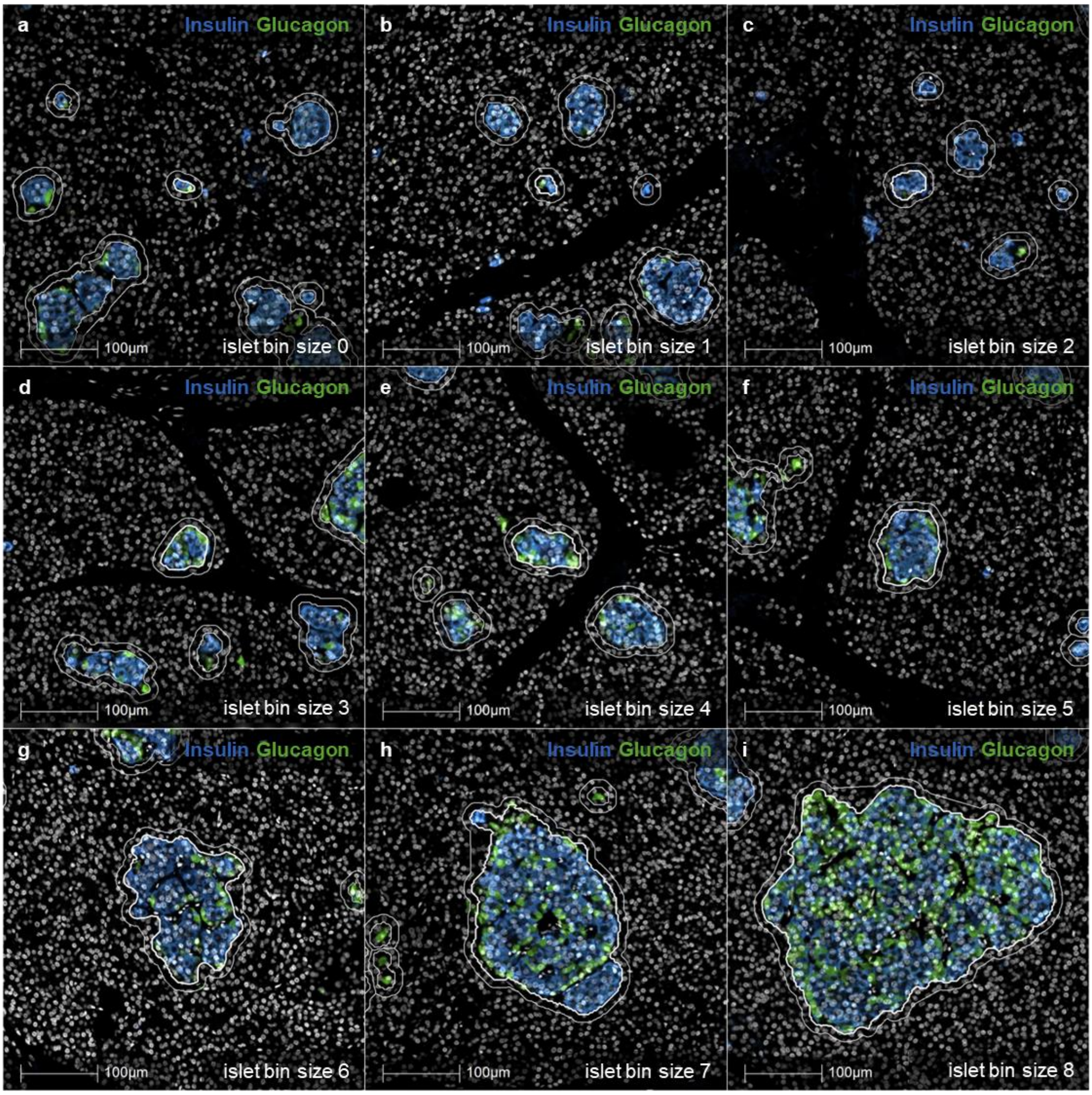

ESM Fig. 2 Representative islets from each islet bin size category.
White annotations show islets and islet periphery (10 µm outside of the islet), the bold white annotation in each micrograph highlights the islet which corresponds to the listed islet bin size. Donor ID: 6501, Individual ≥13 years without type 1 diabetes, nPOD.

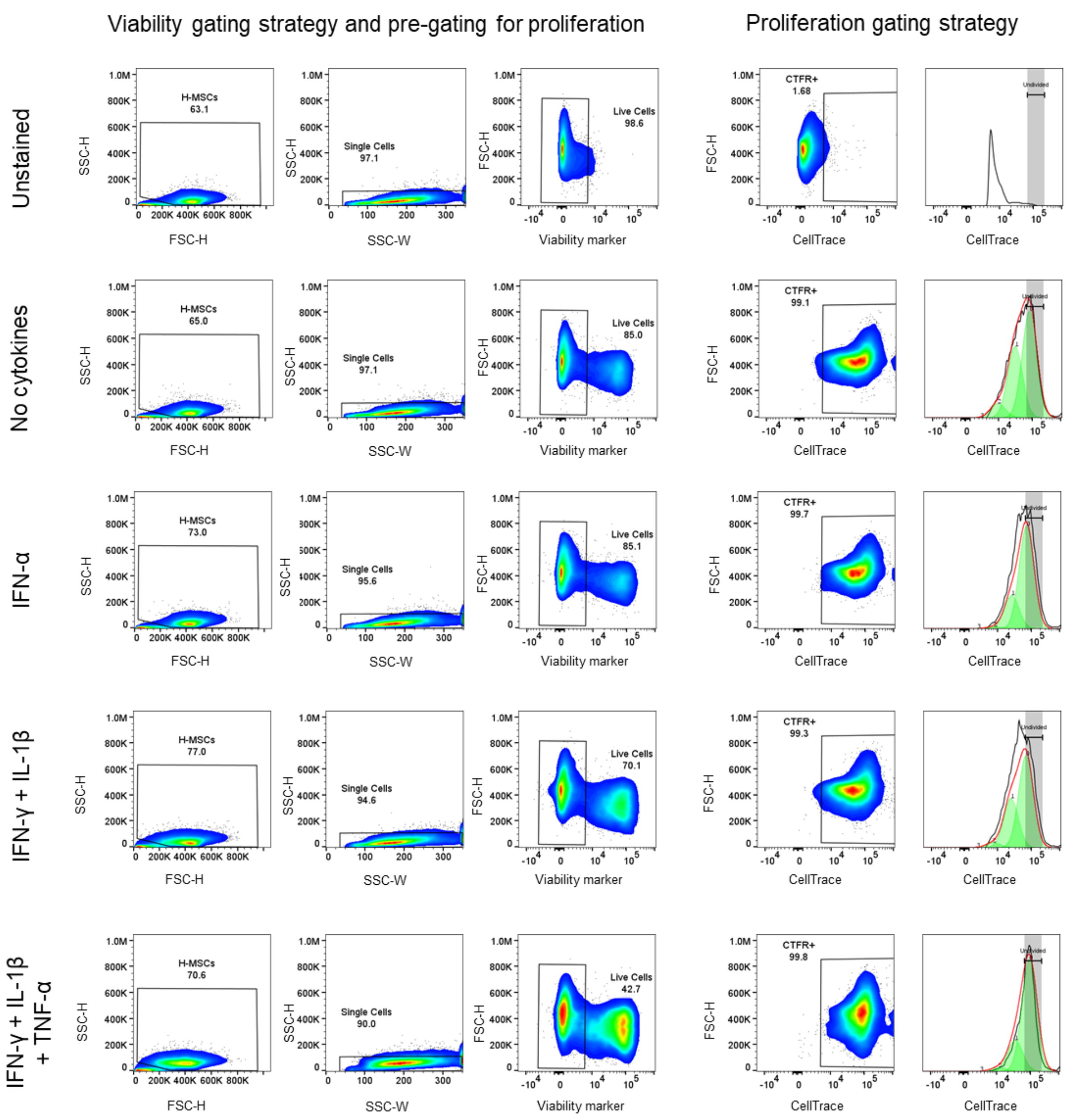

ESM Fig. 3 Gating strategy for in vitro viability and proliferation of cytokine-exposed MSCs. All representative figures from one independent experiment following three days cytokine exposure. Grey highlighted area within proliferation gating section represents gating for the undivided cell population.

CTFR, CellTrace far red; FSC-H, forward scatter height; H-MSC, human mesenchymal stromal cell; SSC-H, side scatter hight; SSC-W, side scatter width.

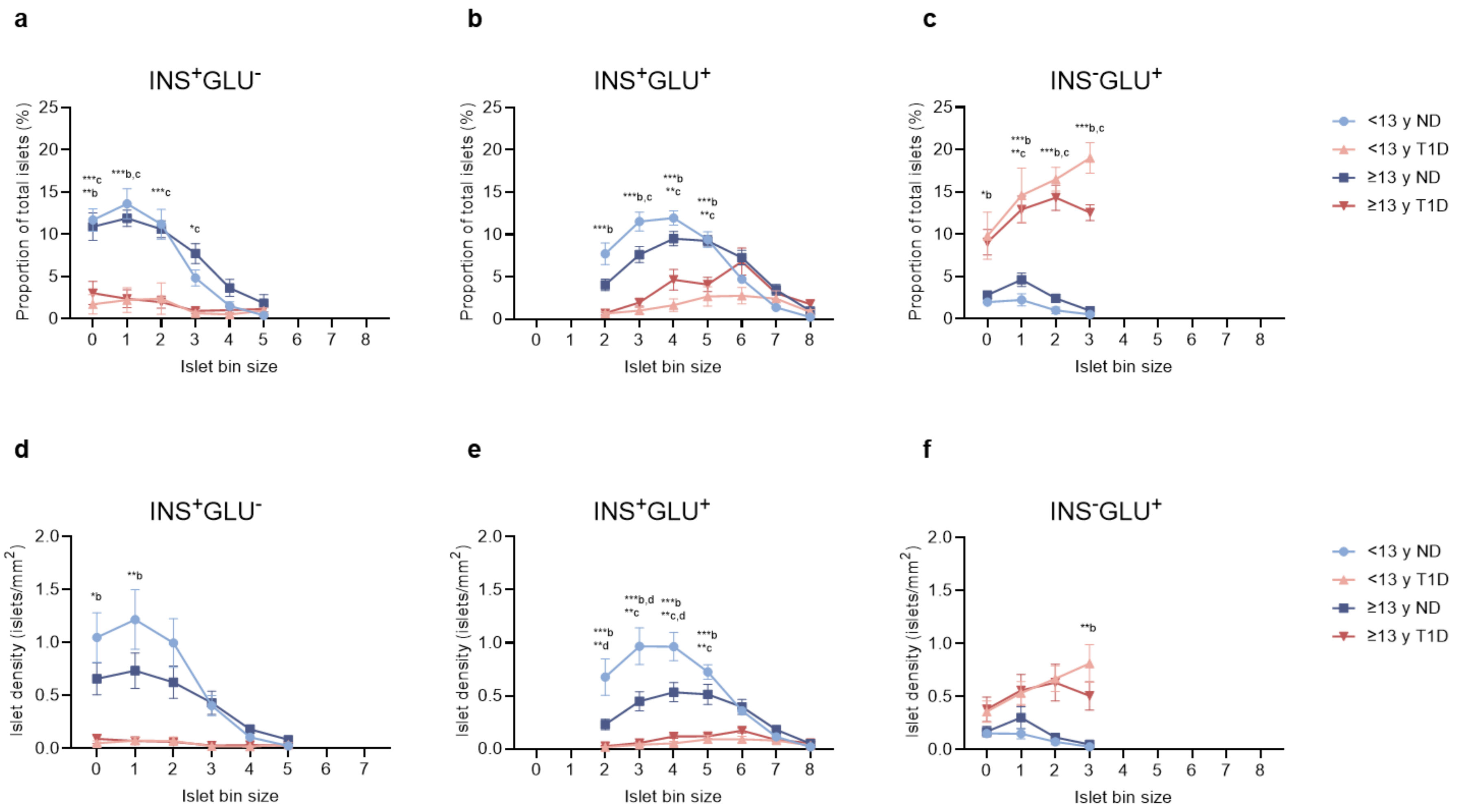

ESM Fig. 4 Density and proportion of islets comprising each endocrine cell composition, separated by islet bin size.
Islet bin sizes containing <5 islets, or bin sizes devoid of data for one or more group were excluded; data derived from *n*=23,290 individual islets.
Bars represent mean ± SEM. Two-way ordinary ANOVA with post hoc tests adjusted for multiple comparisons with Bonferroni correction.

Post hoc comparisons within each islet bin size in **a-f**: <13 years at type 1 diabetes diagnosis vs <13 years without diabetes (‘[b]’); ≥13 years at type 1 diabetes diagnosis vs ≥13 years without diabetes (‘[c]’); <13 years without diabetes vs ≥13 years without (‘[d]’)’.
***$p\leq0.001$; **$p\leq0.01$; *$p\leq0.05$.
<13 y ND, <13 years without diabetes; <13 y T1D, <13 years at type 1 diabetes diagnosis; ≥13 y ND, ≥13 years without diabetes; ≥13 y T1D, ≥13 years at type 1 diabetes diagnosis.

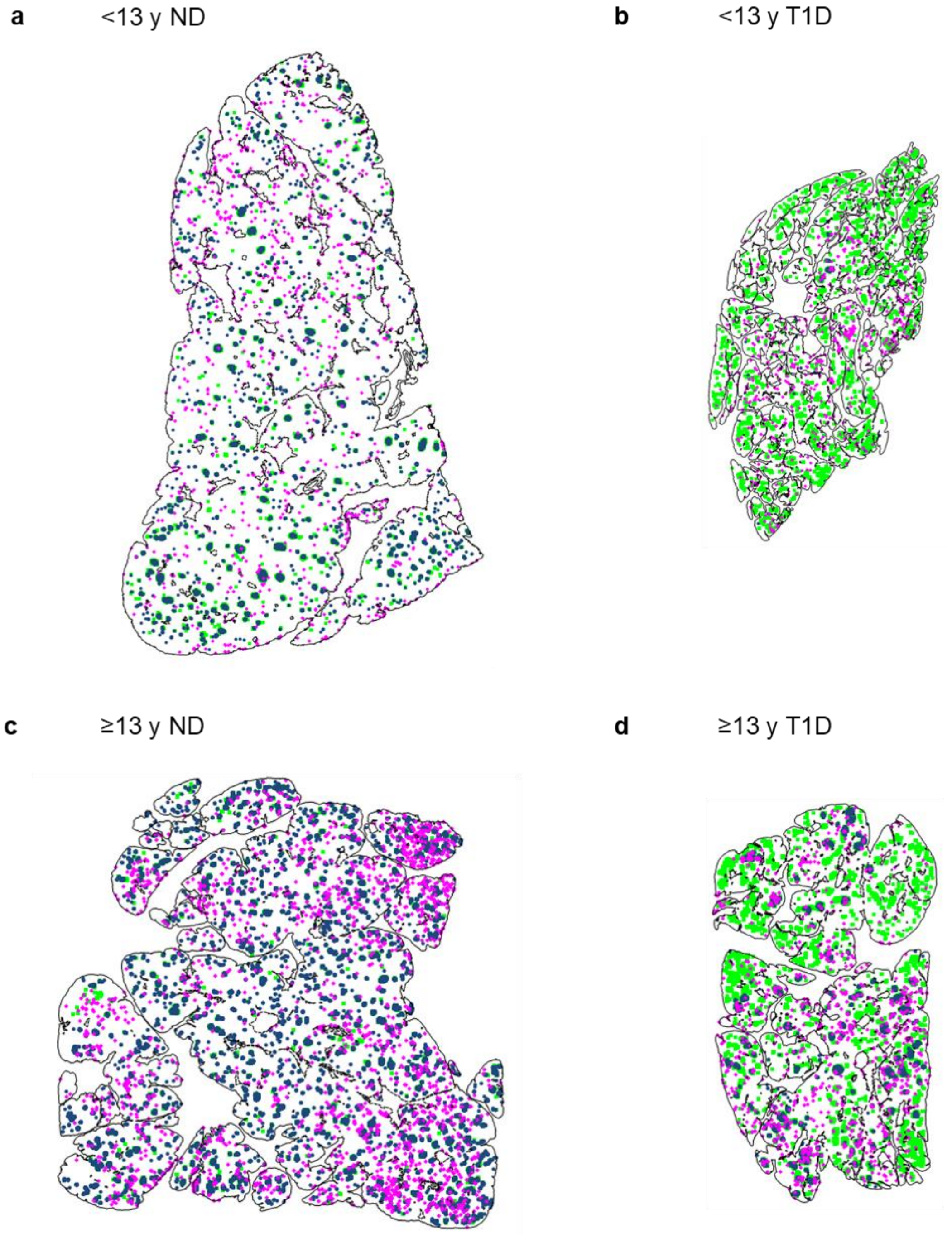

ESM Fig. 5 Distribution of pMSCs across the pancreas.

Representative spatial plots generated in HALO showing pMSC distribution across the pancreas. Pink diamonds show individual MSCs, green squares show alpha cells (glucagon^{+} cells), and dark blue circles show beta cells (insulin^{+} cells). The black outline indicates the pancreas area.

Donor IDs: nPOD 6407 (**a**), nPOD 6533 (**b**), nPOD 6339 (**c**), nPOD 6362 (**d**).

<13 y ND, <13 years without diabetes; <13 y T1D, <13 years at type 1 diabetes diagnosis; ≥13 y ND, ≥13 years without diabetes; ≥13 y T1D, ≥13 years at type 1 diabetes diagnosis.

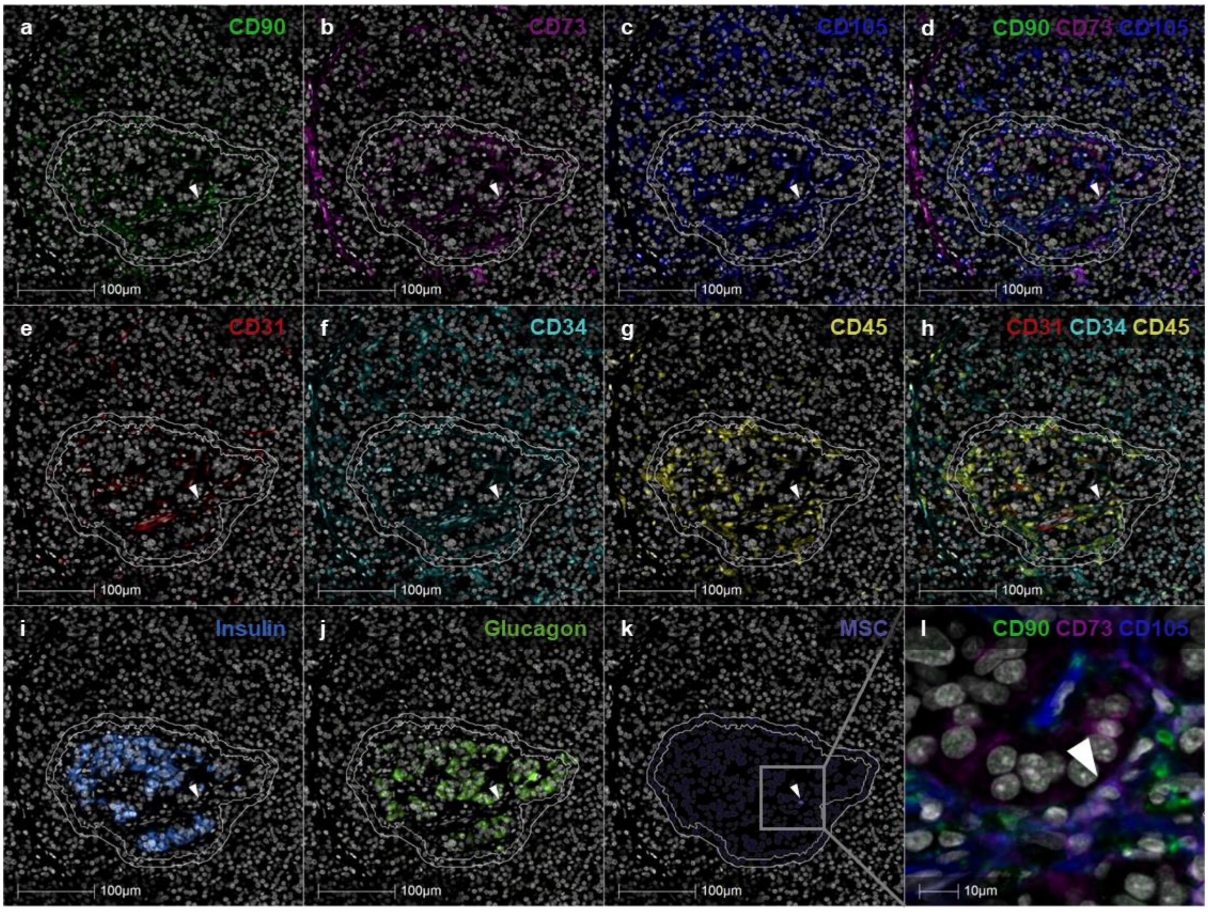

ESM Fig. 6 Phenotype and morphology of intraislet pMSCs.

Intraislet pMSCs (shown by white arrows; **a-l**) were identified in HALO as CD90⁺ (**a**), CD73⁺ (**b**), CD105⁺ ((**c**); overlay of positive markers, (**d**)), CD31⁻ (**e**), CD34⁻ (**f**), CD45⁻ ((**g**); overlay of negative markers, (**h**)). Islets were identified by insulin (**i**) and glucagon (**j**) immunostaining (inner white annotation) and the islet annotation was expanded by 10 µm or until another annotation was reached (outer white annotation). Next, pMSCs were quantified (**k**). A magnified micrograph of **k** is shown in **l** (grey box shows magnified area) and intraislet pMSCs were identified (**l**).

Micrographs used in the representative figure were adjusted to optimise contrast and visibility without altering the underlying image data or quantification. Adjustments were made to the ‘Black In’, ‘White In’ and ‘Gamma’ settings.

Donor ID: <13 years at type 1 diabetes diagnosis, 6578, nPOD.

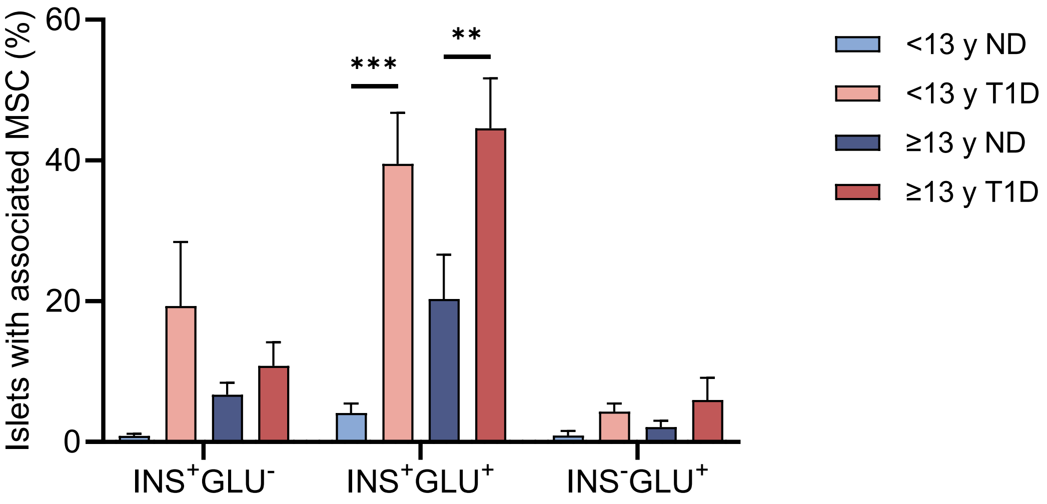

ESM Fig. 7 The proportion of islets with an associated pMSC is increased for insulin-containing glucagon-containing islets in type 1 diabetes.

Islets with one or more pMSC either inside the islet or within 10 µm of the islet periphery were defined as having an associated pMSC.

Data derived from *n*=26,376 individual islets from 38 individuals (*n*=8 individuals <13 years at type 1 diabetes diagnosis and *n*=11 individuals ≥13 years at type 1 diabetes diagnosis; *n*=8 individuals <13 years without diabetes and *n*=11 individuals >13 years without diabetes).

Bars represent mean ± SEM. Two-way ordinary ANOVA with post hoc tests adjusted for multiple comparisons with Bonferroni correction. Post hoc comparisons: type 1 diabetes group and individuals of similar age without diabetes; type 1 diabetes and age of diagnosis; individuals without diabetes. Comparisons shown with a horizontal black line between the two compared groups.

***$p \leq 0.001$; **$p \leq 0.01$

<13 y ND, <13 years without diabetes; <13 y T1D, <13 years at type 1 diabetes diagnosis; ≥13 y ND, ≥13 years without diabetes; ≥13 y T1D, ≥13 years at type 1 diabetes diagnosis.

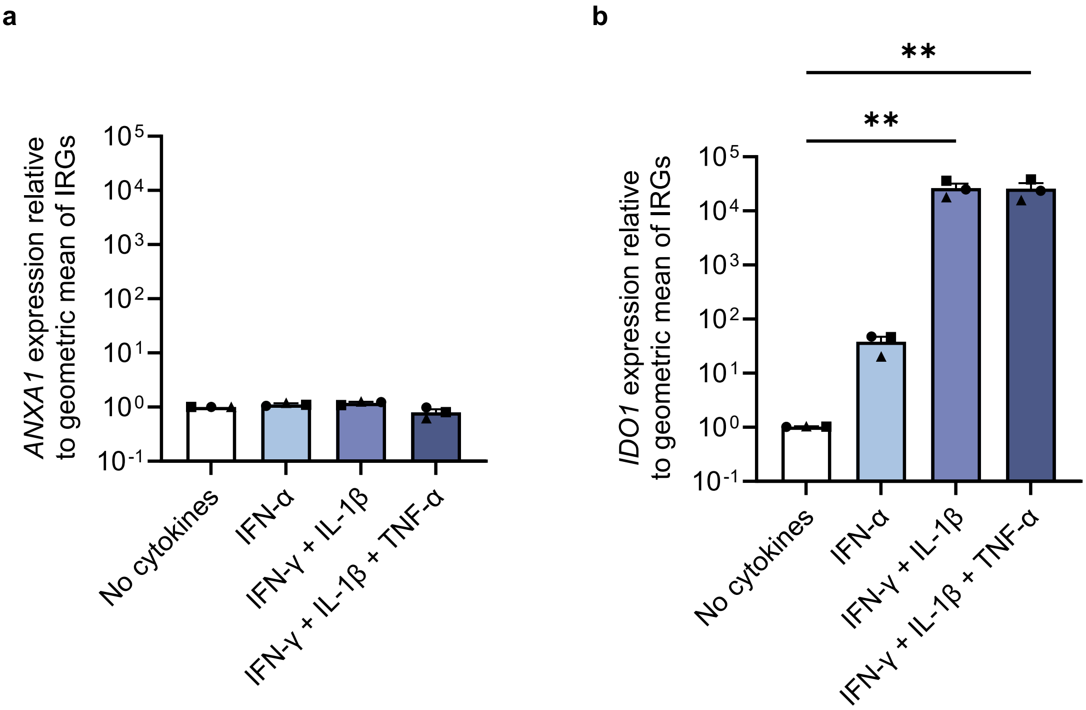

ESM Fig. 8 MSC gene expression of the islet-protective factors *ANXA1* and *IDO1* following cytokine exposure in vitro.

ANXA1 (**a**) and *IDO1* (**b**) gene expression relative to the geometric mean of the internal reference genes *ACTB*, *GAPDH*, *PPIA* and *HPRT1*. Data shown as relative changes to the expression of *ANXA1* and *IDO1* relative to the no cytokine condition.

Each symbol represents an independent experiment (n=3). Bars represent mean ± SEM. One-way ANOVA with post hoc tests using the no cytokine group as the comparator. Post hoc tests adjusted for multiple comparisons with Bonferroni corrections. Comparisons shown with a horizontal black line between the two compared groups.

**$p \le 0.01$.

IRG, internal reference gene.
